# Microglia Regulate Blood–Brain Barrier Integrity via MiR‐126a‐5p/MMP9 Axis during Inflammatory Demyelination

**DOI:** 10.1002/advs.202105442

**Published:** 2022-06-27

**Authors:** Zhongwang Yu, Xue Fang, Weili Liu, Rui Sun, Jintao Zhou, Yingyan Pu, Ming Zhao, Dingya Sun, Zhenghua Xiang, Peng Liu, Yuqiang Ding, Li Cao, Cheng He

**Affiliations:** ^1^ Institute of Neuroscience Key Laboratory of Molecular Neurobiology of Ministry of Education and the Collaborative Innovation Center for Brain Science SMMU Shanghai 200433 China; ^2^ Department of Gastroenterology Changhai Hospital SMMU Shanghai 200433 China; ^3^ Department of Neurology Changhai Hospital SMMU Shanghai 200433 China; ^4^ Department of Laboratory Animal Science and State Key Laboratory of Medical Neurobiology and MOE Frontiers Center for Brain Science Institutes of Brain Science Fudan University Shanghai 200032 China

**Keywords:** blood–brain barrier, experimental autoimmune encephalomyelitis, microglia, miR‐126a‐5p, multiple sclerosis

## Abstract

Blood–brain barrier (BBB) impairment is an early prevalent feature of multiple sclerosis (MS), and remains vital for MS progression. Microglial activation precedes BBB disruption and cellular infiltrates in the brain of MS patients. However, little is known about the function of microglia in BBB impairment. Here, microglia acts as an important modulator of BBB integrity in inflammatory demyelination. Microglial depletion profoundly ameliorates BBB impairment in experimental autoimmune encephalomyelitis (EAE). Specifically, miR‐126a‐5p in microglia is positively correlated with BBB integrity in four types of MS plaques. Mechanistically, microglial deletion of miR‐126a‐5p exacerbates BBB leakage and EAE severity. The protective effect of miR‐126a‐5p is mimicked and restored by specific inhibition of MMP9 in microglia. Importantly, Auranofin, an FDA‐approved drug, is identified to protect BBB integrity and mitigate EAE progression via a microglial miR‐126a‐5p dependent mechanism. Taken together, microglia can be manipulated to protect BBB integrity and ameliorate inflammatory demyelination. Targeting microglia to regulate BBB permeability merits consideration in therapeutic interventions in MS.

## Graphical abstract

Microglial depletion mitigates blood–brain barrier (BBB) disruption during inflammatory demyelination. MiR‐126a‐5p in microglia is positively correlated with BBB integrity in four types of multiple sclerosis plaques. Microglia ameliorates BBB destruction via MiR‐126a‐5p/MMP9 axis. Auranofin elevates miR‐126a‐5p to preserve BBB integrity and alleviate experimental autoimmune encephalomyelitis progression.

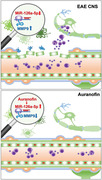

## 1 Introduction

The blood–brain barrier (BBB) is a highly specialized structure in the central nervous system (CNS). As a physical and chemical barrier, the BBB selectively separates the brain parenchyma from the peripheral blood cells, neurotoxic plasma components and pathogens, as well as regulates influx and efflux transport of molecules to maintain brain homeostasis.^[^ ^1^, ^2^ ^]^ Composed of vascular endothelial cells, astrocytes, pericytes, and supported by extracellular matrix, the BBB has continuous tight junctions and basal membrane, lacking fenestrations.^[^ ^3^ ^]^ Previous studies have found that hypoxia, inflammation, tumors, infection, and other environmental factors can disrupt BBB integrity.^[^ ^3^, ^4^, ^5^ ^]^ BBB impairment is an early feature of many CNS pathologies.^[^ ^2^, ^3^, ^6^ ^]^

Multiple sclerosis (MS) is the most prevalent chronic inflammatory demyelinating disease of the CNS.^[^ ^7^ ^]^ Most lesions in MS are perivascular.^[^ ^7^ ^]^ BBB disruption starts earlier than 90% of MS symptoms and can be observed even in normal‐appearing white matter around lesion sites, represented by a decrease in tight junction proteins and leakage of plasma components.^[^ ^8^ ^]^ In addition, dynamic contrast‐enhanced MRI analysis indicates BBB permeability is intricately linked to MS relapse activity.^[^ ^9^ ^]^ However, the mechanisms underlying the regulation of BBB integrity in MS remain elusive.^[^ ^10^, ^11^, ^12^, ^13^, ^14^ ^]^

Microglia are the CNS‐resident immune cells, and their activation is one of the earliest features of MS.^[^ ^15^ ^]^ Accumulating evidence supports the vital role of microglia in MS,^[^ ^16^, ^17^, ^18^ ^]^ either through antigen presentation, complement activation, or interactions with neurons, astrocytes, and oligodendrocytes. While the roles of pericytes, smooth muscle cells, vascular endothelial cells, astrocytes and oligodendroglia on the BBB have been recognized, how microglia regulate BBB integrity in MS remains elusive.^[^ ^8^, ^19^, ^20^ ^]^ It is reported that microglia have little effect on BBB integrity under physiological conditions,^[^ ^21^, ^22^ ^]^ whereas microglial activation lead to BBB disruption during systemic inflammation or ischemic stroke.^[^ ^23^, ^24^ ^]^ In marmoset EAE, BBB impairment with attendant microglial activation was prior to peripheral inflammatory cell infiltration that accompanies demyelination.^[^ ^25^ ^]^ However, whether and how microglia regulate BBB permeability during inflammatory demyelination is largely unknown.

In this study, we sought to investigate the roles of microglia on BBB integrity in the pathogenesis of MS and EAE. We revealed that the removal of microglia significantly mitigated BBB disruption in EAE mice. We further demonstrated that microglial miR‐126a‐5p alleviated BBB destruction by MMP9 inhibition. Auranofin, an FDA‐approved drug, was identified to effectively preserve BBB integrity and ameliorate EAE progression by elevating miR‐126‐5p expression. Our results suggest microglia may represent a possible therapeutic target for MS treatment.

## 2 Results

### 2.1 Microglial Depletion Alleviates BBB Destruction and Retards EAE Progression

**Figure 1:**
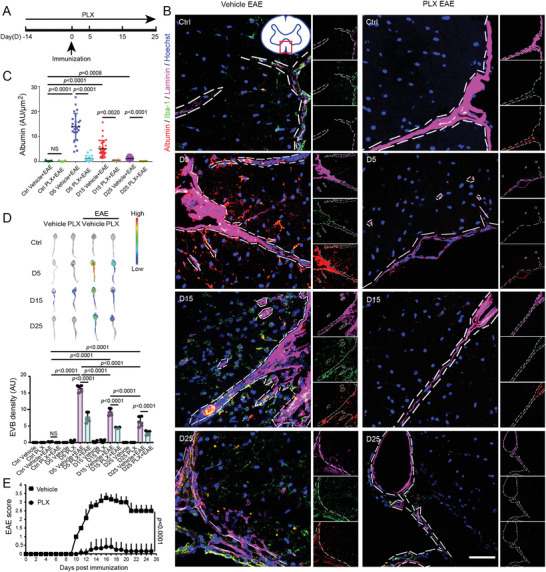
Microglial ablation attenuates BBB impairment and ameliorates EAE progression. A) Scheme of microglial ablation and time points for immunization and examination. B) Temporal dissemination of the BBB in the lumbar spinal cord from EAE mice following PLX5622 administration. Representative images of Iba‐1 (green), Albumin (red), Laminin (magenta, Alexa Fluor 647), and cell nuclei (blue) showing the integrity dynamic development in coronal sections of spinal cords from EAE mice at the early (Day 5 post immunization, D5), middle (Day 15 post immunization, D15), and late stages (Day 25 post immunization, D25). Naïve mice are used as a control group (Ctrl). The dashed lines indicate the boundary of Laminin^+^ vessels. Inset: Spinal cord section with a box denoting observed area. Scale bar: 50 µm. C) Quantitative analysis of relative intensity of Albumin in each group as indicated in (B). Kruskal–Wallis test with Dunnett's multiple comparisons test is used. D) Representative images showing BBB integrity in vehicle or PLX5622 treated mice with or without EAE after *i.v*. EVB injection by optical imaging. One‐way ANOVA with a Holm–Sidak's multiple comparisons test is used. *N* = 3 mice at each time point per group for immunofluorescence staining and EVB extravasation assay. E) The clinical score of EAE administrated with PLX5622 or vehicle daily from 14 d before immunization to 25 d post immunization. Mann–Whitney test is used. *N* = 9 mice per group for EAE score analysis. Data are shown as the mean ± SEM.

To investigate the role of microglia on BBB integrity in MS progression, we used the CSF1R inhibitor PLX5622 to eliminate microglia in EAE mice (**Figure** **1**A). Histopathological study revealed that the microglia in the lumbar spinal cord of mice was activated no later than 5 d post immunization (D5). Microglial activation was accompanied by albumin exudation, reflective of aggravation of vascular leakage. At D15, there were hypercellularity indicating extensive infiltration by inflammatory cells, concomitant with a large number of microglia accumulated in the area of albumin exudation (Figure 1B,C). These data suggested that the early activation of microglia was associated with the impairment of the BBB prior to the infiltration of inflammatory cells. To further study BBB impairment in EAE, we examined Evans blue dye (EVB) extravasation. Our results showed that BBB integrity was most severely damaged on D5, followed by slight recovery at D15 and D25 (Figure 1D). Consistent with previous observations in EAE,^[^ ^26^ ^]^ the peak of BBB destruction (D5) was earlier than the onset of clinical symptoms (D10) (Figure 1E).

Intragastric administration of PLX5622 at dose of 90 mg kg^−1^ d^−1^ led to sustained depletion of microglia in CNS (Figure 1B). Compared with that in the control group (Vehicle+EAE group), microglial depletion (PLX+EAE group) significantly decreased albumin leakage throughout the entire course of EAE (Figure 1B,C). Similarly, microglial ablation attenuated EVB extravasation in EAE mice, suggesting the amelioration of BBB impairment (Figure 1D). Consequently, the severity of EAE was significantly reduced after microglia depletion in EAE mice (Figure 1E), consistent with Nissen's observation.^[^ ^27^ ^]^ Furthermore, the EAE score was still lower than that in control group when the mice were treated with PLX5622 at the dosage of 67.5 mg kg^−1^, or even 45 mg kg^−1^ (Figure S1A,B, Supporting Information). We also investigated the effect of microglial depletion by giving 90 mg kg^−1^ PLX5622 after the peak period of BBB destruction (D5) during EAE progression (PLX5622‐B group, Figure S1C, Supporting Information). The PLX5622‐B group was less effective to ameliorate EAE progression as compared to that with PLX5622 pretreatment (PLX5622‐A group, Figure S1D, Supporting Information). Taken together, these results indicated that early removal of microglia could effectively alleviate BBB impairment and reduce the EAE severity.

Previous studies demonstrated that PLX5622 also acts on other CSF‐1R^+^ cells, including monocytes and macrophages, though with minimal effects.^[^ ^28^, ^29^ ^]^ To circumvent the issue, we used CX3CR1^CreER^:iDTR mice (CX3CR1‐DTR) to ablate microglia and then examined BBB integrity during EAE progression. In CX3CR1‐DTR mice, four weeks after tamoxifen induction, at the time of immunization, peripheral macrophages or monocytes had renewed and no longer expressed DTR, leaving microglia being eliminated after Diphtheria toxin (DT) injection (Figure S2A–C, Supporting Information). In contrast, the YFP^+^ microglia in control mice (CX3CR1‐Ctrl) during the same period were preserved (Figure S2D, Supporting Information).

DT ablation effects lasted at least 3 d, and microglia repopulation might start no later than 7 d.^[^ ^22^, ^30^ ^]^ Thus, after 4 d of the last DT injection (0.1 µg/mouse for three times), PLX5622 was administrated to keep microglial depletion (Figure S2A, Supporting Information). To interrogate the specific of target cell types in CX3CR1‐DTR system, additional FACS analyses are performed. Virtually all CX3CR1‐YFP^+^ CD45^int^ CD11b^+^ cells are validated to be Ly6C^–^ in FACS analysis, and they are bona fide microglia. Significant depletion of microglia (defined as CX3CR1‐YFP^+^ CD45^int^ CD11b^+^ Ly6C^−^) were observed in CX3CR1‐DTR group (CX3CR1‐DTR 4.07% ± 0.12% vs CX3CR1‐Ctrl 38.13% ± 5.26%, *p* < 0.0001), indicating the majority population deleted 24 h after DT injection (EAE D2) are primarily microglia (Figure S3, Supporting Information). We further ruled out the possibility that decrease of microglia in CX3CR1‐DTR group was attributed to microglial activation into CX3CR1‐YFP^+^ CD45^high^ CD11b^+^ Ly6C^−^, given that most CX3CR1‐YFP^+^ CD45^high^ CD11b^+^ cells is Ly6C^+^. By contrast, no synchronized reduction was observed on EAE D2 in peripheral immune cell populations, defined as CX3CR1‐YFP^+^ CD45^high^ CD11b^+^ Ly6C^+^ cells (CX3CR1‐DTR 37.21% ± 4.07% vs CX3CR1‐Ctrl 25.74% ± 5.56%, *p* = 0.1151), ruling out the possibility that the meliorate CNS pathology was attributed to the decrease of peripheral immune cells infiltration. Further investigation revealed that, on EAE D6 (Figure S4, Supporting Information), 24 h after subsequent PLX treatment, substantial depletion of microglia were observed in CX3CR1‐DTR group (CX3CR1‐DTR 5.02% ± 0.15% vs CX3CR1‐Ctrl 39.48% ± 0.37%, *p* < 0.0001). Whereas, no synchronized decrease was observed in peripheral immune cell populations (CX3CR1‐DTR 6.30% ± 0.78% vs CX3CR1‐Ctrl 4.56% ± 0.39%, *p* = 0.0749). Altogether, the above lines of evidence support predominant microglial depletion using the CX3CR1‐DTR system without grossly decrease peripheral immune cell infiltrates.

Immunofluorescence staining showed that the albumin exudation was remarkably decreased following the ablation of microglia (Figure S2D,E, Supporting Information). Consistently, the EVB extravasation assay (Figure S2F,G, Supporting Information) showed that BBB destruction was significantly attenuated by microglial depletion. The progression of EAE was also ameliorated in CX3CR1‐DTR group (Figure S2H, Supporting Information). Therefore, genetically removal of microglia could also preserve BBB integrity and ameliorate EAE progression.

### 2.2 Mir‐126a‐5p in Microglia Is Inversely Correlated with BBB Impairment during Inflammatory Demyelination

**Figure 2:**
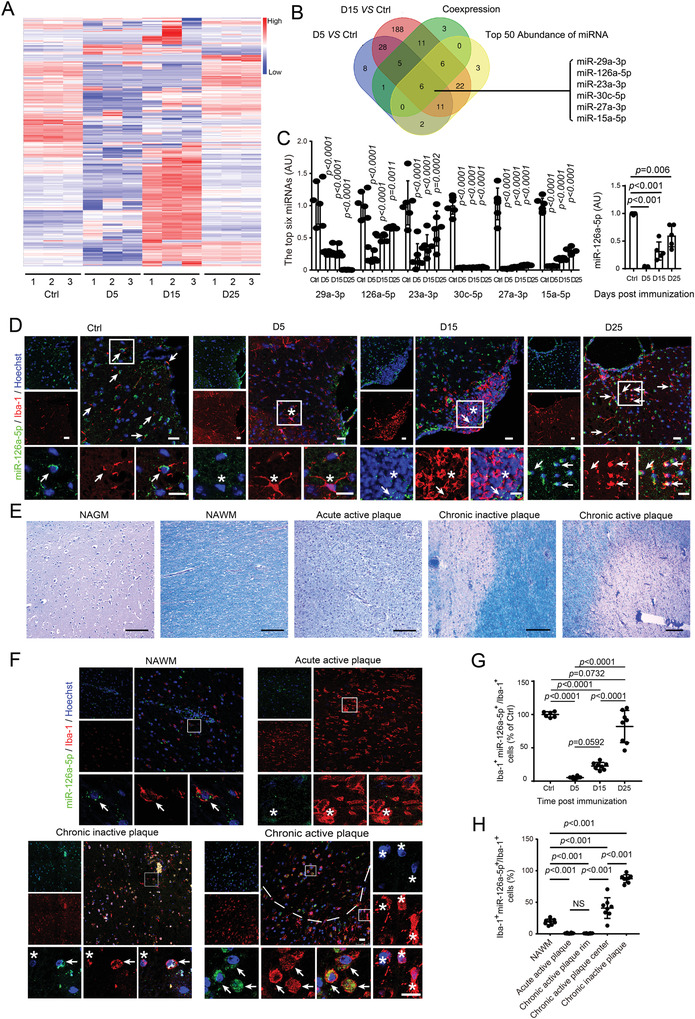
Microglial miR‐126a‐5p is identified inversely correlated with BBB impairment in EAE and MS. A) Heatmap of expression profiles of miRNA in FACS sorting microglia from spinal cord of EAE mice at indicated time points depicts up‐ or downregulated miRNA as compared with Ctrl group (Naive microglia). *N* = 3 independent replicates (each containing five mice per group). B) The Venn diagram depicting the miRNAs that overlap four conditions: the differential miRNA in the early (1) or middle (2) stage group versus Ctrl, (3) co‐expression analysis, and (4) the TOP 50 in the abundance hierarchies of miRNA. C) The selected six miRNAs are validated. One‐way ANOVA with a Holm–Sidak's multiple comparisons test is used. *P*‐value is indicated versus each Ctrl group. Left: *N* = 6 per group. Right: *N* = 5 per group. D) Representative image of miR‐126a‐5p in different stages of EAE. Arrows indicate Iba‐1^+^ miR‐126a‐5p^+^ cells. Stars indicate Iba‐1^+^ miR‐126a‐5p^–^ cells. Scale bars: 20 µm. *N* = 3 mice per group. E) Histology analysis of MS lesion using LFB‐PAS staining. Scale bars: 50 µm. *N* = 8 per group. F) Representative image of miR‐126a‐5p in different MS lesion sites. Note that more Iba‐1^+^ miR‐126a‐5p^+^ cells (arrows) in the center of chronic active plaque, while more Iba‐1^+^ miR‐126a‐5p^−^ cells (stars) in the rim (dashed lines) nearby. Scale bars: 20 µm. *N* = 8 per group. G) Quantitative analysis of Iba‐1^+^ miR‐126a‐5p^+^/Iba‐1^+^ cells in lumbar spinal cords in EAE. *N* = 3 mice per group. H) Quantitative analysis of Iba‐1^+^ miR‐126a‐5p^+^/Iba‐1^+^ cells in MS. N = 8 per group. One‐way ANOVA with a Tukey's multiple comparisons test is used in (G, H). Data are shown as the mean ± SEM.

MiRNAs play critical roles during the pathogenesis of MS. While MS‐associated regulation of miRNAs have been reported in many different cell types, their function on BBB integrity remains enigmatic.^[^ ^31^ ^]^ To explore microglial mechanisms of BBB disruption during inflammatory demyelination, we compared the miRNomes of sorted microglia from EAE mice before and after the peak of BBB destruction (**Figure** **2**A). We selected the time points in the EAE progression based on previous studies,^[^ ^32^ ^]^ which suggest day 5 as early stage, day 15 as middle stage, and day 25 as late stage.

BBB impairment in EAE mice experienced a process of aggravation and then partially remission (Figure 1B). To screen for microglial miRNA associated with BBB integrity, the miRNAs profile of FACS sorted microglia on the early stage (D5) (Set 1) and the middle stage (D15) (Set 2), were compared with the control group (Ctrl), respectively (Figure 2B). We chose miRNAs that were synchronous with the pattern of EVB extravasation (|R| > 90%, *P* < 0.05) as set 3, and the top 50 abundance of miRNAs in microglia as set 4 (see also Table S1, Supporting Information) (Figure 2B). Consequently, we identified six overlapping miRNAs (Figure 2B), which had the highest abundance, significant difference, and similar expression pattern in line with the permeability change of the BBB. We performed QPCR verification for these miRNAs (Figure 2C). Specifically, we found the expression of miR‐126a‐5p was transiently reduced at D5 followed by an incomplete recovery (Figure 2A,C). Therefore, the level of miR‐126a‐5p in microglia in EAE mice was inversely correlated with BBB impairment that was peaked within the first 5 d and was partially rescued during the following days (Figure 1B,C).

To further characterize miR‐126a‐5p expression, we performed fluorescence in situ hybridization (FISH) analysis. Consistently, the results illustrated that miR‐126a‐5p levels were significantly downregulated in microglia in the spinal cord at D5, and gradually recovered at D15 and D25 (Figure 2D,G).

Considering the conservation of miRNA, we also detected the expression pattern of miR‐126a‐5p in MS patient brain slices (Figure 2E). Compared with normally appearing white matter (NAWM), miR‐126a‐5p levels were extremely low in acute active lesions, but significantly increased in microglia in chronic inactive lesions. We also found that the percentage miR‐126a‐5p^+^ microglia in the center of the chronic active lesion (remission area of BBB damage) was higher than that in the rim of lesions (Figure 2F,H).

### 2.3 Deficiency of Mir‐126a‐5p in Myeloid Cell Increases BBB Permeability and Exacerbates EAE Progression

To evaluate the function of microglial miR‐126a‐5p in BBB integrity during EAE progression, we used myeloid cells specific knockout mice (LysM^Cre^:miR‐126^fl/fl^, LysM‐126KO) (Figure S5A, Supporting Information). As expected, the level of miR‐126a‐5p in Iba‐1 positive microglia of the spinal cord was significantly reduced (Figure S5B, Supporting Information). EVB extravasation results showed that BBB impairment in LysM‐126KO mice was more severe than control EAE mice (LysM^Cre^:miR‐126^+/+^, LysM‐Ctrl) (Figure S5C, Supporting Information). Using albumin immunostaining, we also found that BBB impairment was more severe in the LysM‐126KO group than in the control group (Figure S5E, Supporting Information). Interestingly, the albumin exudation in the LysM‐126KO group seemed remarkable in the early stage (Inserts in Figure S5E, Supporting Information). Accordingly, the EAE score of LysM‐126KO mice was higher than that of the control group (Figure S5D, Supporting Information). Moreover, miR‐126a‐5p knockout in myeloid cell augmented inflammatory cell infiltration and demyelination (Figure S5F,G, Supporting Information), showing by LFB‐PAS and FluoroMyelin staining. These results suggest that miR‐126a‐5p in myeloid cells is necessary for protecting BBB integrity and alleviating EAE progression.

### 2.4 MiR‐126a‐5p Deficiency in Microglia Exacerbates BBB Disruption and EAE Progression

**Figure 3:**
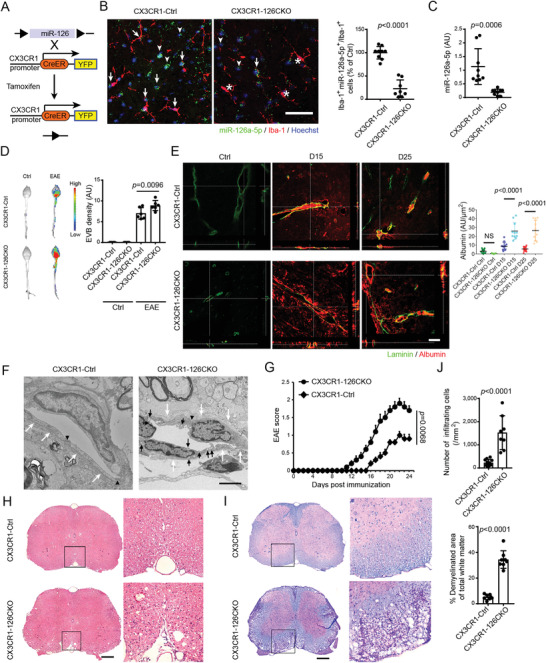
Ablation of miR‐126a‐5p in microglia exacerbates BBB leakage and EAE progression. A) Schematic graph of generation of microglial specific miR‐126a‐5p conditional knockout mice. B,C) The miR‐126a‐5p expression in microglia is determined by FISH and QPCR, respectively. Arrows indicate Iba‐1^+^ miR‐126a‐5p^+^ cells. Arrow heads indicate Iba‐1^−^ miR‐126a‐5p^+^ cells. Stars indicate Iba‐1^+^ miR‐126a‐5p^−^ cells. Scale bars: 50 µm. In (B): *N* = 9 fields for control (CX3CR1^CreER^:126^+/+^, CX3CR1‐Ctrl) group; *N* = 8 fields for conditional knockout (CX3CR1^CreER^:126^fl/fl^, CX3CR1‐126CKO) group. In (C): *N* = 3 mice per group. D) Representative images show BBB integrity in CX3CR1‐Ctrl or CX3CR1‐126CKO mice with or without EAE on D15 after *i.v*. EVB injection by optical imaging. *N* = 3 mice per group. E) Representative orthogonal projections of confocal *z*‐axis stacks show the leakage of Albumin (red) beyond the vessel boundary (Laminin, green) in the lumbar spinal cord. Scale bar: 50 µm. *N* = 3 mice per group. F) EM analysis of BBB on D15. White arrows, basement membrane. Arrowheads, tight junctions between adjacent endothelial cells. Black arrows, vacuolization of endothelial cells with the adjacent tissue severe edematous. Scale bar: 2 µm. *N* = 3 mice per group. G) Clinical scores of EAE mice. *N* = 10 mice for CX3CR1‐Ctrl group. *N* = 11 mice for CX3CR1‐126CKO group. H,I) Representative spinal cord sections from EAE mice after H&E and LFB‐PAS staining. Scale bars: 200 µm. J) Histology analysis of the extent of infiltration and demyelination. *N* = 3 mice per group for both staining. Unpaired Student's *t*‐test is used in (B, C, J). One‐way ANOVA with Holm–Sidak's multiple comparisons test is used in (D, E). Mann–Whitey test is used in (G). Data are shown as the mean ± SEM.

A variety of myeloid cells have been reported to be involved in MS development.^[^ ^17^, ^33^ ^]^ To further understand the specific role of microglial miR‐126a‐5p in EAE, we used CX3CR1^CreER^: miR‐126^fl/fl^ (CX3CR1‐126CKO) mice to delete miR‐126a‐5p specifically in microglia (**Figure** **3**A). The level of miR‐126a‐5p was indeed reduced in Iba‐1^+^ microglia in spinal cord 28 d after tamoxifen induction (Figure 3B,C). EVB extravasation assay showed that the BBB of CX3CR1‐126CKO EAE mice was impaired more severely than the CX3CR1‐Ctrl EAE mice (Figure 3D). There was no remarkable destruction of the BBB in non‐EAE CX3CR1‐126CKO mice (Figure 3D), suggesting that microglial miR‐126a‐5p might play critical roles to maintain BBB integrity under pathological conditions. In addition, albumin extravasation, was more significant in the CX3CR1‐126CKO group than that in the CX3CR1‐Ctrl group, especially in the late stage (Figure 3E). Electron microscopy analysis revealed that the basement membrane became discontinuous with the formation of vacuoles in CX3CR1‐126CKO group, compared to that in the control group. Additionally, the tight junctions between adjacent endothelial cells were separated from the capillary lumen to the basement membrane in CX3CR1‐126CKO mice (Figure 3F). Consistently, the EAE score of CX3CR1‐126CKO mice was higher (Figure 3G), with more cell infiltration and demyelination than those of the control group (Figure 3H–J), showing by hematoxylin and eosin (H&E) and LFB‐PAS staining. These results indicated that microglial miR‐126a‐5p deficiency exacerbates BBB destruction and EAE progression.

Astrocytic endfeet, endothelial cells, pericytes, together with microglia, form a neurovascular unit that constitutes BBB.^[^ ^34^ ^]^ We further supervised any change of these components in our study. As illustrated by staining for Aqp4, a water channel highly enriched in astrocytic endfeet at the BBB, microglial ablation of miR‐126a‐5p group exhibited an increased disruption of astrocytic endfeet, which was ameliorated by microglial depletion, both using pharmacological and genetic approaches (Figure S6, Supporting Information). We also concluded that microglial ablation of miR‐126a‐5p led to decrease of CD31^+^ endothelial activation, whilst microglial depletion rescued it (Figure S6, Supporting Information). Microglial ablation of miR‐126a‐5p induced concurrent PDGFR*β* ^+^ pericytes deficits, which was ameliorated by microglial depletion (Figure S7, Supporting Information).

### 2.5 The Mir‐126a‐5p in Microglia Is Crucial for Preserving the Barrier Integrity of Endothelial Cells In Vitro

To explore the direct effect of miR‐126a‐5p in microglia on BBB integrity, we performed paracellular permeability assay by using cultured human umbilical vein endothelial cells (HUVECs) in transwell inserts with the conditional medium (CM) from microglia. We found that the CM from microglia infected with lentivirus encoding miR‐126a‐5p‐interfering sequence (miR‐126a‐5p‐i) resulted in increased diffusion of fluorescein isothiocyanate (FITC)‐dextran (Figure S8A, Supporting Information). On the contrary, the CM from microglia infected with lentivirus encoding miR‐126a‐5p sequence (miR‐126a‐5p‐OE) had a protective effect on the barrier, showing by lower fluorescence intensity in the diffusate (Figure S8A, Supporting Information). We also measured the transendothelial electrical resistance (TEER) of the endothelial monolayer, as higher resistance indicating a tighter barrier.^[^ ^35^ ^]^ The electrical impedance of the barrier decreased remarkably in the miR‐126a‐5p‐i group, suggesting the destruction of the barrier integrity (Figure S8B, Supporting Information). On the contrary, the electrical impedance of the barrier increased in the miR‐126a‐5p‐OE group, indicating the maintained barrier integrity. The immunofluorescence staining (Figure S8C,D, Supporting Information) and immunoblot assay (Figure S8E,F, Supporting Information) further verified increased expression of ZO‐1 and occludin (OCLN) in the miR‐126a‐5p‐OE group, and decreased expression of these tight junction proteins in the miR‐126a‐5p‐i group. To corroborate the function of miR‐126a‐5p in BBB integrity, we further used cultured mouse brain microvascular endothelial cells (BMVECs). Consistently, the TEER of BMVECs was lower in the miR‐126a‐5p‐i group and higher in the miR‐126a‐5p‐OE group, exhibiting the destruction and protection of the barrier integrity, respectively (Figure S8G, Supporting Information). In addition, we manipulated miR‐126a‐5p in microglia by miR‐126a‐5p mimics. The CM from microglia incubated with miR‐126a‐5p mimics could significantly increase the level of ZO‐1 in BMVECs (Figure S8H–J, Supporting Information), further confirming that miR‐126a‐5p in microglia maintains the barrier integrity. Taken together, these results demonstrate that microglial miR‐126a‐5p is crucial for protecting the barrier integrity of endothelial cells in vitro.

### 2.6 MMP9 Servers as the Target of Mir‐126a‐5p in Microglia

To investigate the mechanism underlying the effect of microglial miR‐126a‐5p, we first measured production of reactive oxygen species (ROS), interleukin‐1*β* (IL‐1*β*), tumor necrosis factor (TNF‐*α*), and nitric oxide (NO) in microglia that are known to regulate BBB integrity.^[^ ^3^, ^36^ ^]^ The results showed that manipulating miR‐126a‐5p in microglia did not affect the levels of these inflammatory mediators (Figure S9, Supporting Information).

**Figure 4:**
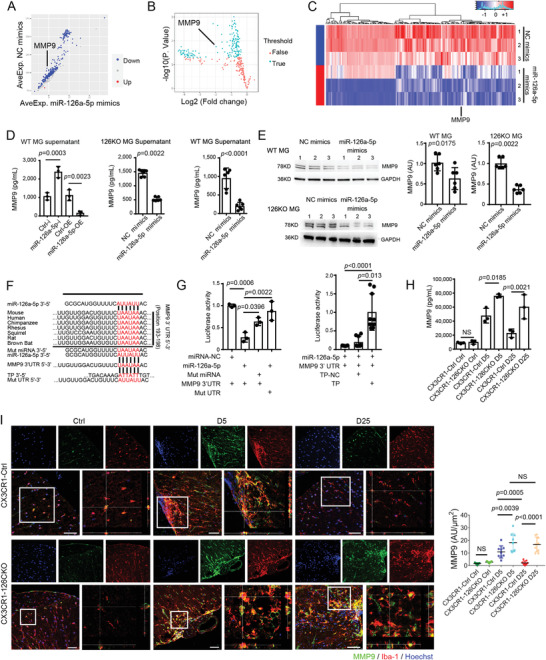
MMP9 serves as the target of miR‐126a‐5p in microglia. A) Scatter plot, B) volcano plot, and C) heat map illustrate differentially expressed proteins in microglia between miR‐126a‐5p mimics and NC mimics groups. *N* = 3 per group. D) MMP9 levels in microglia supernatant by ELISA. *N* ≥ 3 per group. E) Immunoblot and densitometric analyses of MMP9 in microglia as indicated. *N* = 6 per group. F) Top: The putative miR‐126a‐5p binding sites in 3' UTR of MMP9 mRNA among species. Bottom: Sequence alignment of miR‐126a‐5p, target protector (TP) or mutant miRNA (Mut miRNA) with the MMP9 3' UTR or mutated mRNA 3' UTR (Mut UTR). G) Left: Luciferase activity of reporter carrying the MMP9 3' UTR or mutated mRNA 3' UTR (Mut UTR) co‐transfected into HEK293T cells with miR‐126a‐5p mimics, mimics control (miRNA‐NC) or mutant miRNA (Mut miRNA). *N* = 3 per group. Right: Luciferase activity of reporter in HEK293T cells co‐transfected with the MMP9 mRNA 3' UTR and miR‐126a‐5p mimics carrying TP or Ctrl (TP‐NC). *N* = 9 per group. (H) MMP9 levels in the cerebrospinal fluid of indicated EAE mice by ELISA. *N* = 3 per group. I) Representative images of MMP9 in microglia in the lumbar spinal cord of indicated EAE mice. Orthogonal projections of *z*‐axis stacks show co‐localization of MMP9 and Iba‐1 in microglia. Scale bars: 50 µm. *N* = 9 per group. One‐way ANOVA with Holm–Sidak's multiple comparisons test is used in (D, G, H, I). Unpaired Student's *t*‐test or Mann–Whitney test is used in (D, E). Kruskal–Wallis test with Dunnett's multiple comparisons test is used in (G). Data are shown as the mean ± SEM.

To identify the potential target(s) of miR‐126a‐5p in microglia, we performed the protein array assay to detect the supernatant of microglial lysate from the LysM‐126KO microglia transfected with miR‐126a‐5p mimics (miR‐126a‐5p mimics) or NC mimics (control mimics) (**Figure** **4**A–C; Table S2, Supporting Information). By comparing the differential protein profile between the two groups and analyzing the target genes of miR‐126a‐5p based on target prediction websites (TargetScan, TarBase, RNAhybrid), five overlapping molecules (matrix metalloproteinase 9, Sonic Hedgehog, E‐selectin, C reactive protein, and Toll‐like receptor 4) were identified. Among them, we selected matrix metallopeptidase 9 (mmp9), which is both a microglia‐specific highly expressed^[^ ^37^ ^]^ and the most significantly changed gene (logFC = −1.59, *p* = 0.011, Table S2, Supporting Information), as a candidate target of miR‐126a‐5p to do further investigation.

To determine the relationship between miR‐126a‐5p and mmp9 in vitro, we performed ELISA of supernatant from wild type microglia (WT MG) or microglia from LysM‐126KO mice (126KO MG). The results showed that miR‐126a‐5p upregulation (miR‐126a‐5p‐OE or miR‐126a‐5p mimics) reduced the level of MMP9, whereas miR‐126a‐5p downregulation (miR‐126a‐5p‐i) increased the level of MMP9 (Figure 4D). In addition, Western blotting further confirmed that miR‐126a‐5p mimics significantly reduced the level of MMP9 in the microglia from both wild‐type and 126KO mice (Figure 4E). Therefore, miR‐126a‐5p is capable to regulate the expression of MMP9 in microglia.

To examine whether miR‐126a‐5p could directly interact with mmp9 gene, we performed luciferase reporter assay. As shown in Figure 4F, there is a potential binding site for miR‐126a‐5p in 3' UTR of mmp9, which is highly conserved among different species. Indeed, the miR‐126a‐5p specifically interacted with the 3' UTR site of mmp9 to suppress the luciferase activity, as either mutation on the sequence of miR‐126a‐5p or on the 3' UTR would relieve the suppression (Figure 4G). Additionally, the LNA‐modified target protector (TP) could relieve the repression of 3' UTR of mmp9 by miR‐126a‐5p (Figure 4G). Taken together, these results suggest miR‐126a‐5p exerts inhibitory effect on mmp9 expression by binding to its 3' UTR.

Next, we tested whether miR‐126a‐5p regulates the expression of MMP9 in vivo. As shown by ELISA (Figure 4H), the cerebrospinal fluid (CSF) of CX3CR1‐126CKO mice during EAE exhibited a surge of MMP9 compared with that of control (CX3CR1‐Ctrl) mice. Moreover, immunohistochemistry study showed persistently higher levels of MMP9 in microglia of CX3CR1‐126CKO mice than those in the non‐knockout group in EAE progression. These results indicate that the deficiency of miR‐126a‐5p in microglia results in a high level of MMP9 in the CNS of EAE mice.

### 2.7 MMP9 Is Indispensable for BBB Impairment Induced by Microglial Mir‐126a‐5p Deficiency

**Figure 5:**
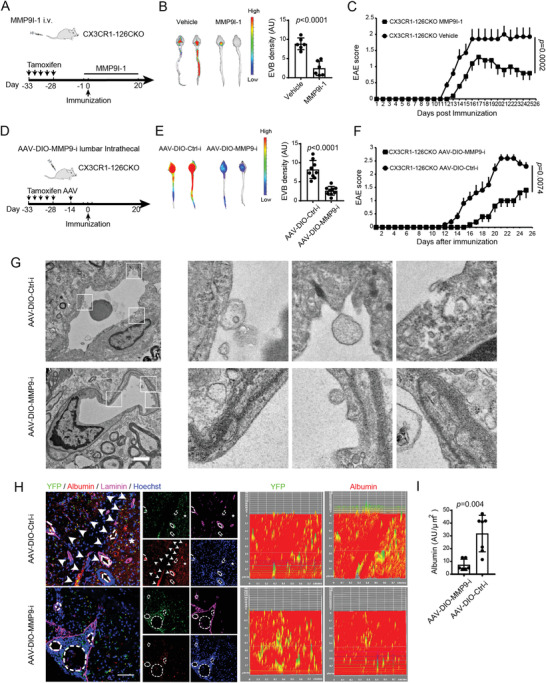
MMP9 is indispensable for BBB impairment induced by the microglial ablation of miR‐126a‐5p. A) Scheme of microglial ablation of miR‐126a‐5p and administration of MMP9 inhibitor (MMP9I‐1). B) Representative images show BBB integrity in CX3CR1‐126CKO EAE mice with or without MMP9I‐1 administration on D25. *N* = 3 mice per group. C) Clinical score of CX3CR1‐126CKO EAE mice treated with MMP9I‐1. *N* = 5 for vehicle. *N* = 7 for MMP9I‐1. D) Scheme of microglial specific MMP9 inhibition in CX3CR1‐126CKO EAE mice. E) Representative images show BBB integrity in CX3CR1‐126CKO EAE mice with or without cell specific MMP9 inhibition on D25. *N* = 3 mice per group. F) Clinical score of CX3CR1‐126CKO EAE mice transfected with DIO‐MMP9‐i‐AAV. *N* = 5 per group. G) EM analysis of the BBB in the CX3CR1‐126CKO mice with or without microglial MMP9 inhibition. The boxes in the left panels are enlarged in the right panels. The basement membrane is discontinuous in AAV‐DIO‐Ctrl‐i group, with some tight junctions separated and loosed. On the contrary, the continuous basement membrane and the tight junctions are well reserved in AAV‐DIO‐MMP9‐i group. Scale bar: 2 µm. *N* = 3 per group. H) Representative images of YFP, albumin, laminin, and Hoechst show BBB integrity in lumbar spinal cord sections at D25. Note the boundary (arrowheads) of albumin leakage (Star) and the boundary of Laminin^+^ vessels (dashed lines). I) Quantitative analysis of relative intensity of albumin in (H). *N* = 6 for AAV‐DIO‐MMP9‐i group. *N* = 7 for AAV‐DIO‐Ctrl‐i group. Unpaired Student's *t*‐test is used in (B, E). Mann–Whitney test is used in (C, F). Data are shown as the mean ± SEM.

To examine the role of MMP9 in the miR‐126a‐5p dependent BBB impairment during EAE, we first performed pharmacology study. CX3CR1‐126CKO mice were given MMP9 inhibitor I‐1 (MMP9I‐1) before and after MOG immunization (**Figure** **5**A). The EVB extravasation assay showed that, compared with vehicle group, BBB impairment was alleviated under the treatment of MMP9I‐1 (Figure 5B). The EAE score of CX3CR1‐126CKO mice was also decreased in the MMP9I‐1 treated group (Figure 5C).

Next, we constructed a Cre‐driven AAV to genetically downregulate MMP9 (AAV‐DIO‐MMP9‐i). After depletion of MMP9 in microglia by intrathecal injection of AAV‐DIO‐MMP9‐i, the CX3CR1‐126CKO mice were immunized with MOG (Figure 5D). We found that BBB leakage in the AAV‐DIO‐MMP9‐i group was greatly alleviated compared with that in the control group (AAV‐DIO‐Ctrl‐i) (Figure 5E), while the EAE score in the AAV‐DIO‐MMP9‐i group was significantly reduced (Figure 5F).

Electron microscopy analysis indicated that the basement membrane of vascular endothelial in the lumbar spinal cord in the AAV‐DIO‐MMP9‐i group was well reserved (Figure 5G). By contrast, the basement membrane in the AAV‐DIO‐Ctrl‐i group was discontinuous, accompanied by loosed or separated tight junctions. The albumin exudation in the lumbar spinal cord in AAV‐DIO‐MMP9‐i group was consistently reduced in CX3CR1‐126CKO mice 25 days postimmunization (Figure 5H,I).

### 2.8 Auranofin Alleviates BBB Impairment and EAE Progression via Microglial Mir‐126a‐5p Dependent Protection

**Figure 6:**
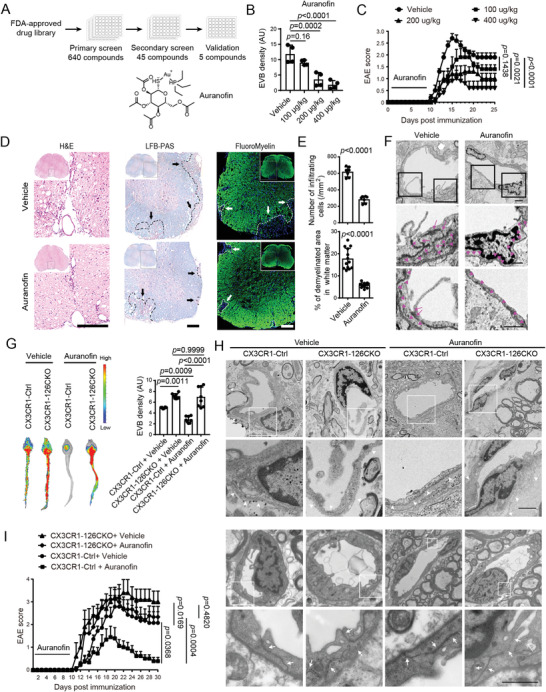
Auranofin alleviates BBB impairment and EAE progression via surging miR‐126a‐5p in microglia. A) Drug screen work flow and structural formula of Auranofin. B) EVB density of EAE mice on D15. *N* = 4 per group. C) Clinical score of Auranofin‐treated EAE mice. *N* = 5 per group. D) Representative spinal cord sections from EAE D15 mice treated with Auranofin (200 µg kg^−1^) after H&E, LFB‐PAS, or FluoroMyelin staining. Arrows indicate demyelination. Scale bars: 200 µm. E) Quantification of infiltration and demyelination in EAE mice on D15. *N* ≥ 7 per group. F) EM analysis of the BBB in EAE mice with or without Auranofin treatment. The boxes in the upper panel are enlarged in the lower panels. The basement membrane (arrowheads) and tight junctions (arrows) are reserved in Auranofin‐treated group, whereas basement membrane becomes coarse in the vehicle group, with some tight junctions separated and scrambled. Scale bar: 1 µm. *N* = 3 per group. G) EVB density of EAE mice on D15. *N* = 8 per group. H) EM analysis of the BBB in EAE mice on D15. The boxes in the upper panels are enlarged in the lower panels. Note the perivascular spaces are widened irregularly, with generalized rarefaction of basement membranes (arrowheads) in the CX3CR1‐126CKO + Auranofin group. Even in samples which reserve continuous basement membrane, the tight junctions (arrows) are partially separated. Scale bar: 1 µm. *N* = 3 per group. I) Clinical score of EAE mice. *N* = 7 for CX3CR1‐Ctrl+Vehicle. *N* = 9 for CX3CR1‐Ctrl+ Auranofin. *N* = 5 for CX3CR1‐126CKO+ Vehicle. *N* = 9 for CX3CR1‐126CKO+Auranofin. Kruskal–Wallis test with Dunn's multiple comparisons is used in (C, I). One‐way ANOVA with Holm–Sidak's multiple comparisons test is used in (B, G). Data are shown as the mean ± SEM.

To test whether the BBB impairment during inflammatory demyelination could be rescued by induction of miR‐126a‐5p, we screened an FDA‐approved drug library containing 640 small molecule compounds. In the primary screen, we identified 45 compounds which were ranked into four tiers on the basis of miR‐126a‐5p level in microglia (**Figure** **6**A; Table S3, Supporting Information). After the secondary screen, we further selected the top five candidates, based on the average fold change of miR‐126a‐5p as compared with control (Table S3, Supporting Information) (Figure 6A). Then, we performed additional validation to detect mmp9 level in microglia treated with each of these candidates (Table S4, Supporting Information) (Figure 6A). Among them, Auranofin, which has been used as an anti‐rheumatoid arthritis,^[^ ^38^ ^]^ antiparasitic and anticancer agent,^[^ ^39^ ^]^ exerted a salient effect on elevation of miR‐126a‐5p and reduction of mmp9 simultaneously. Thus, we next examined the effect of Auranofin on EAE mice. The EVB extravasation assay revealed that both 200 and 400 µg kg^−1^ Auranofin effectively preserved BBB integrity (Figure 6B). In addition, Auranofin treatment, starting from the beginning (Figure 6C) but not from D10 (Figure S10, Supporting Information), could greatly ameliorate the severity of EAE, which was further corroborated by the reduced demyelination and cell infiltration (Figure 6D,E). Consistent with the result in EVB extravasation assay, EM analysis indicated that Auranofin preserved the integrity of the basement membrane of vascular endothelial cells in the lumbar spinal cord parenchyma of EAE mice (Figure 6F). We further examined the effect of Auranofin on EAE mice with microglial miR‐126a‐5p deficiency. The EVB extravasation assay and EM analysis demonstrated that Auranofin treatment lost the beneficial effect on preserving BBB integrity in the CX3CR1‐126CKO mice (Figure 6G,H). Consistent with these observations, Auranofin also failed to alleviate the severity of EAE in the CX3CR1‐126CKO mice, whereas the control EAE mice were significantly improved after the treatment (Figure 6I). Taken together, Auranofin protected BBB integrity and mitigated EAE progression via a microglial miR‐126a‐5p dependent mechanism.

## 3 Discussion

MS is the most common chronic inflammatory demyelinating disease of the CNS. Although MS is considered to be initially triggered by perturbation of peripheral immune responses, microglia are known to be crucial in the onset and progression of the disease.^[^ ^40^ ^]^ Recently, 551 putative causal risk loci for MS derived from comprehensive MS genetic reference map have been identified, most of which are enriched in microglia.^[^ ^17^ ^]^ Previous studies demonstrate multiple roles of microglia in MS and EAE, including inflammation induction,^[^ ^41^ ^]^ synapse removal,^[^ ^42^ ^]^ and remyelination.^[^ ^43^, ^44^, ^45^ ^]^ In the present study, we provide strong evidence that the microglia impair BBB integrity during the early stage of EAE, implying the detrimental role of microglia activation in the onset of MS.

BBB permeability is intricately linked to the progress and relapse of MS.^[^ ^9^ ^]^ As previously reported,^[^ ^25^ ^]^ the cell infiltration and BBB destruction are most obvious in the acute lesions; whereas the chronic inactive plaques exhibit well‐demarcated borders with few or no inflammatory cells, indicating a partially restored BBB integrity. In addition, the chronic active MS lesions are a mixture, consisting of compromised BBB permeability in the rim and restored BBB integrity in the center. However, the mechanism underlying the regulation of BBB integrity in the lesions remains enigmatic.

Actually, our observation of microglia depletion under normal condition is consistent to what has been previously described,^[^ ^46^ ^]^ i.e., it has no detectable impact on overall vascular permeably. Notably, we further validated microglia is deleterious to vascular structure during inflammatory demyelination, and microglia depletion ensure BBB integrity under this condition. It is likely that the effect of microglia has changed from vasodilation under physiological condition^[^ ^46^ ^]^ to vacuolization of endothelial cells with the adjacent tissue severe edematous, recapitulating vascular leakage, under pathophysiological condition. Besides, it was also reported that microglia enhance BBB leakage^[^ ^47^ ^]^ or mediate resealing of leaks^[^ ^48^ ^]^ in BBB in stroke. Taken together, our results and previous studies support microglia play different roles on vascular structure and BBB under physiological and pathophysiological conditions.

To date, a variety of miRNAs have been identified in MS lesions with different activities, though little is known on the expression pattern of miRNAs in specific cells in the lesions.^[^ ^49^ ^]^ Our data demonstrate that miR‐126a‐5p is highly expressed in microglia in chronic inactivated MS lesions and in the center of chronic active MS lesions, consistent with previous studies that miR‐126 in chronic inactive lesions is 2.4 times to normal brain white matter although the cell type with upregulated miR‐126 was not appreciated.^[^ ^49^ ^]^ Thus, our results strongly suggest that the level of mir‐126a‐5p in microglia is highly correlated with the degree of BBB deterioration in human MS lesions, which is reminiscent of its potential causal relationship with BBB integrity in MS.

Notably, maintaining the integrity of the BBB is a necessary prerequisite for relieving early stage of EAE and acute MS, but in the later stage of EAE or in the chronic inactive MS lesion, when the change of BBB permeability becomes less obvious, other factors, such as cleaning up inhibitory molecule for remyelination may be more critical in parallel.^[^ ^50^ ^]^ For instance, though exposure of endothelium to proinflammatory cytokines (TNF‐alpha and IL‐1beta) interrupts the BBB,^[^ ^51^ ^]^ several challenges of anti‐IL‐1b treatment still exist^[^ ^52^ ^]^ and anti‐TNF*α* by infliximab^[^ ^53^, ^54^, ^55^ ^]^ even worsen progressive MS. Recent data suggest that anti‐TNF*α* therapy has a potential role on the induction of demyelination of the CNS, while IL‐1*β* can actually promote remyelination.^[^ ^56^, ^57^ ^]^ These studies indicate an insufficient role of preserving BBB integrity in later stage or in chronic plaques to alleviate progressive MS. Likewise, in our study, while starting Auranofin treatment from EAE D0 suppresses BBB leakage, enhancing miR‐126a‐5p in microglia is insufficient to exert therapeutic effects when Auranofin is given after EAE D10. What indicates that, despite alleviateing BBB leakage in the later stage of EAE, benefit of enhancing miR‐126a‐5p to remyelination seems limited. Nevertheless, we emphasize the timing of attenuating BBB leakage is critical. To reserve the BBB integrity via maintaining sufficient miR‐126a‐5p in microglia on the early stage, is important to alleviate later demyelination.

To clarify the function of miR‐126a‐5p in microglia, we specifically deleted miR‐126a‐5p in microglia. Notably, despite the BBB in the miR‐126 knockout mice remained intact under physiological conditions, BBB impairment was exaggerated during EAE. Previous studies indicated that miR‐126 could promote endothelial proliferation in coronary artery disease^[^ ^58^ ^]^ and atherosclerosis,^[^ ^59^ ^]^ increase smooth muscle cell turnover,^[^ ^60^ ^]^ modulate angiogenesis in physical and pathological conditions.^[^ ^61^ ^]^ In addition, miR‐126 was shown to control the self‐renewal of hematopoietic stem cells in leukemia^[^ ^62^ ^]^ and regulate primitive erythropoiesis.^[^ ^63^ ^]^ In the present study, we demonstrate that the miR‐126a‐5p in microglia is crucial for preserving BBB integrity during inflammatory demyelination. Our study highlights a novel function of miR‐126a‐5p specifically in microglia which merits consideration for therapeutic modulation of MS.

Coordinated regulation of normal neurovascular functions depends on the vascular cells (endothelium and pericytes) and astrocytes,^[^ ^64^ ^]^ which are considered to be triad of BBB. Among them, endothelial cells are connected with each other through the tight junction, adherens junction and gap junction proteins, forming the brain capillaries. Pericytes covered the capillaries and share the basement membrane with endothelium. Astrocytic endfeet form the outer layer of the capillaries. Pericytes and astrocytes are critical for the BBB maintenance. Pericytes communicate with the endothelial cells via growth factor or matrix proteins and influence BBB permeability. Astrocytes can modulate expression of tight junction and basement membrane proteins by regulating pericytes function.^[^ ^65^ ^]^ Our study suggests that microglial depletion of miR‐126a‐5p initiated neurovascular cascade during EAE progression, involving severe disruption of astrocytic endfeet, injury of endothelial cells, and deficits of pericytes. By contrast, microglial depletion protects astrocyte, endothelial cells and pericytes during EAE. Yet, considering mutual interaction among pericytes, endothelial cells and astrocytic endfeet,^[^ ^66^ ^]^ whether associated changes were direct consequences or by‐products of microglial ablation of miR‐126a‐5p remains an open question.

A variety of inflammatory mediators have been suggested to impact the tight junction proteins to affect the BBB.^[^ ^36^ ^]^ In addition, extracellular matrix components have been observed to be elevated in multiple sclerosis, specifically at the basement membranes of cerebral vessels.^[^ ^67^ ^]^ Here, we identified that MMP9, an extracellular matrix protease, was suppressed in microglia following upregulating miR‐126a‐5p. Interestingly, the basal MMP9 level is much higher in microglia than in neurons, astrocytes, or oligodendrocytes, and is even more than ten times higher than in endothelial cells.^[^ ^37^ ^]^ Notably, previous reports showed that the level of MMP9 is higher at the rim of chronic active lesions than in the center or in the peri‐plaque white matter.^[^ ^68^ ^]^ These lines of evidence prompted us to consider MMP9 as the primary candidate target for miR‐126a‐5p. Indeed, we revealed that upregulating miR‐126a‐5p in the cultured microglia reduced the expression of MMP9, whereas downregulating miR‐126a‐5p increased the expression of MMP9. Importantly, pharmacologic or genetic inhibition of MMP9 in microglia abrogated the detrimental effects in BBB destruction and EAE progression caused by the miR‐126a‐5p deficiency. Therefore, the miR‐126a‐5p/MMP9 pathway in microglia is essential for the regulation of BBB integrity during inflammatory demyelination.

Notably, our observation is consistent with recent study on systemic inflammation,^[^ ^23^ ^]^ in which microglia were also discovered to be activated ahead of any detectable change in BBB permeability. Interestingly, contrary to adversely influence BBB integrity by microglia ablation as ones assumed,^[^ ^23^ ^]^ in our study, the microglia activation correlated with BBB leakage. In systemic inflammation, as depicted, though microglia initially protected BBB integrity, nevertheless they were overwhelmingly activated and caused BBB disruption during the prolonged inflammation ultimately. To some extent, in our observation, microglia may also play a dual role: Normally, microglia containing low level but sufficient miR‐126a‐5p to depress MMP9, maintain homeostatic function without detectable change in BBB. On early stage of EAE or in acute active plaque, microglia is activated to suppress miR‐126a‐5p, causing the surge of MMP9, exert a detrimental effect on BBB. From middle stage of EAE, in chronic inactivated MS lesions or in the center of chronic active MS lesions, microglia with rebound miR‐126a‐5p suppress MMP9, thus alleviate BBB leakage. Interestingly, we also found that microglial ablation five days post immunization was less effective to alleviate BBB damage than microglia ablation at the very beginning. Moreover, Auranofin treatment, starting from the beginning but not from D10 could greatly ameliorate the severity of EAE, what also indicates the window period for reversing BBB destruction by microglia is in the early stage of EAE. Thus, our results indicated that microglia may play a dual regulatory role on BBB integrity in different stages of MS and EAE, and highlight the necessity of reversing BBB destruction via elevating the miR‐126a‐5p in microglia at the early stage of inflammatory demyelination.

Currently, the medication for MS mainly focuses on immunomodulation.^[^ ^69^ ^]^ The treatments of MS through preserving BBB integrity are still limited. In this study, by screening the library of FDA‐approved drugs, we identified Auranofin was able to upregulate miR‐126a‐5p and decrease mmp9 in the cultured microglia. Thus, we assume Auranofin mitigated BBB leakage and EAE progression through microglial miR‐126a‐5p‐dependent pathways. Auranofin has been used as drugs for anti‐rheumatoid arthritis, antiparasitic and anticancer.^[^ ^38^, ^39^, ^70^ ^]^ Previous study indicated Auranofin could target thioredoxin reductase, an enzyme involved in ROS detoxification.^[^ ^71^ ^]^ However, as we observed no significant difference of ROS in microglia depletion of miR‐126a‐5p, Auranofin might not exert protective effects on BBB integrity via ROS dependent manner. Notably, Auranofin treatment on a different paradigm (daily from D10 to D20) failed to ameliorate EAE severity (see Figure S10, Supporting Information). It further supported that Auranofin confer beneficial effects predominantly via maintaining BBB integrity, rather than through direct neuroprotection or remyelination. In relapsing‐remitting MS (RRMS) and secondary progressive MS (SPMS), disturbance of the BBB as shown by gadolinium enhancement in MRI is a predictor of the occurrence of relapses,^[^ ^72^ ^]^ which suggested that Auranofin might have a potential therapeutic role in preventing the relapse. Since Auranofin is already FDA approved, the translatability of Auranofin on treating RRMS and SPMS merit further investigation. Primary progressive MS (PPMS) is characterized by chronic inflammation with a closed BBB,^[^ ^73^ ^]^ and disturbance of the BBB is not a strong predictor of the development of cumulative impairment or disability in PPMS.^[^ ^72^ ^]^ This supports the idea that variant pathogenetic mechanisms are operative in the occurrence of relapses and in the development of long‐term disability in MS. Taking together, we speculated that Auranofin may not be effective in preventing the development of PPMS.

Indeed, limiting BBB leakage can reduce CNS access for a range of toxic circulating molecules such as inflammatory cytokines and blood factors, which can deteriorate demyelination or are nonpermissive for remyelination. For instance, it is reported that fibrinogen activates BMP signaling in OPCs and inhibits remyelination after vascular damage.^[^ ^74^ ^]^ Interleukin‐1 promotes inflammatory demyelination by suppressing endothelial heme oxygenase‐1 at the BBB.^[^ ^75^ ^]^ Hence, we cannot rule out the possibilities that Auranofin reduces demyelination via the preservation of OPCs and oligodendrocytes as a by‐stander/subsequent effect from BBB protection. Regardless, since disturbance of the BBB is a predictor of the occurrence of relapses,^[^ ^72^ ^]^ considering RRMS and SPMS is characterized by inflammation with a compromised BBB,^[^ ^76^ ^]^ whereas PPMS is characterized by degeneration with a closed blood–brain barrier,^[^ ^73^ ^]^ the translatability of Auranofin on treating MS, especially SPMS and RRMS, merits further investigation. In addition, microglial ablation five days post immunization was less effective to alleviate BBB damage, what also indicates the window period for reversing BBB destruction by microglia is in the early stage of EAE. Thus, our results highlight the necessity of reversing BBB destruction via elevating the miR‐126a‐5p in microglia at the early stage of inflammatory demyelination.

Understanding the mechanism underlying microglia‐BBB interaction during inflammatory demyelination provides novel insights on MS pathogenesis. Based on our results, microglia presumably has no major effect on the homeostasis of BBB integrity under physiological conditions. Alternatively, microglia would disrupt the barrier integrity under certain pathological conditions, such as inflammation. Our study clarifies a microglial miR‐126a‐5p/MMP9 mediated novel mechanism underlying BBB integrity during inflammatory demyelination and suggests microglial targeting for BBB therapeutic intervention in MS progression. Similarly, in Parkinson's disease, Alzheimer's disease, epilepsy, glioma, and trauma, even COVID‐19,^[^ ^77^ ^]^ microglia warrant necessary attention on their interaction with BBB interaction under these pathological conditions.

## 4 Experimental Section

### Animals and Drug Administration

All animal experiments were performed in adherence with the National Institutes of Health Guidelines on the Care and Use of Laboratory Animals and approved by SMMU Committee on Animal Care. *MiR‐126 ^fl/fl^* (ENSMUSG00000065540) mice were generated in the Shanghai Biomodel Organism Science & Technology Development Co., Ltd. *LysM^Cre^* mice were purchased from the Shanghai Biomodel Organism Science & Technology Development Co., Ltd. *LysM^Cre^* hemizygous mice were crossed with *Rosa26‐yfp* heterozygotes to check for recombination efficiency. *LysM^Cre^* were crossed with *miR‐126 ^fl/fl^* and the F1 *LysM^Cre^*: *miR‐126 ^fl/+^* were then backcrossed to *miR‐126 ^fl/fl^* to generate *LysM^Cre^*: *miR‐126 ^fl/fl^* mice (LysM‐126KO). *CX3CR1^CreER^* mice (Jackson Laboratory) were crossed with *miR‐126 ^fl/fl^* to generate *CX3CR1^CreER^: miR‐126^fl/fl^* (CX3CR1‐126CKO) mice. *CX3CR1^CreER^* mice were also crossed with ROSA26‐iDTR (Jackson Laboratory) to generate CX3CR1^CreER^: iDTR mice (CX3CR1‐DTR). Chemical inhibiting MMP‐9 was performed as previously reported.^[^ ^78^ ^]^ MMP‐9 inhibitor I‐1 (Calbiochem, catalog 444278), at the dosage of 0.1 µg g^−1^ body weight, was administrated intravenously via the tail vein during EAE progression every 48 h since 1 d before the MOG immunization. For Cre‐driven AAV virus transduction to interfere with MMP9, AAV virus to interfere with MMP9 (AAV‐DIO‐MMP9i) or control (AAV‐DIO‐Ctrli) were injected into CX3CR1‐126CKO mice intrathecally as previously described.^[^ ^79^, ^80^ ^]^ For Auranofin treatment, mice were treated with auranofin by intraperitoneal injection at indicated dosage (100, 200, or 400 µg kg^−1^) daily, starting from D0 to D10 or from D10 to D20.

### EAE Induction

EAE induction was performed as previously reported.^[^ ^45^, ^81^ ^]^ Briefly, female C57BL/6 mice (8–10 weeks) were subcutaneously immunized with MOG35–55 (100 µg/mouse, GL Biochem) in complete Freund's adjuvant containing heat‐killed *Mycobacterium tuberculosis* (H37Ra strain; Difco). Pertussis toxin (200 ng/mouse, Calbiochem‐EMD Chemicals) in PBS was administered intraperitoneally on days 0 and 2. Mice were blindly examined daily for disease signs by independent researchers and were assigned scores on a scale of 0–5 as follows: 0, no clinical signs; 1, paralyzed tail; 2, paresis; 3, paraplegia; 4, paraplegia with forelimb weakness or paralysis; and 5, moribund state or death.

### Microglia Depletion by PLX5622

The drug was prepared following TargetMol's protocol (No.1303420‐67‐8). Briefly, PLX5622 stock was dissolved in dimethyl sulfoxide (DMSO) at a concentration of 79 mg mL^−1^ (199.79 × 10^−3^ m); 0.5% sodium carboxymethyl cellulose (Sinopharm Chemical Reagent Co. Ltd.) was prepared to make a diluent. On each dosing day, PLX5622 stock was diluted tenfold by adding 1 volume of drug stock (79 mg mL^−1^) in 9 volumes of diluent, making a working solution at 7.9 mg mL^−1^. Vehicle solution was prepared with a mix of diluent and DMSO. To eliminate microglia in EAE model, PLX5622 of indicated concentration was intragastric administrated every day since 14 d before the MOG immunization until sacrificed.

### Optical Imaging of EVB Fluorescence

BBB permeability was determined in vivo as previously reported.^[^ ^26^ ^]^ Briefly, 100 µL of 1% (w/v) Evans blue (Sigma‐aldrich) in PBS (pH 7.4) were injected intravenously into the tail vein of mice on the indicated time point at a dosage of 5 *μ*L g^−1^ body weight. Four hours after injection, mice were anesthetized and perfused with PBS and paraformaldehyde. Brain and spinal cords were excised and placed for optical imaging. The optical planar fluorescence imaging system (Quick View 3000, Bio‐Real, Austria) was used to quantify the extent of EVB dye fluoresces accumulation with the excitation and emission filters set at 655 and 716 nm, respectively.

### Isolation of Microglia Derived from the Spinal Cord by Flow Cytometry

Microglia (CD11b^+^/CD45^int^) were purified as previously reported.^[^ ^45^ ^]^ The spinal cords of normal or EAE mice were isolated, homogenized, filtrated, centrifuged, and suspended in 70% Percoll (GE Healthcare) and overlaid with 37% and 30% gradient. After centrifugation, the majority of mononuclear cells, found in the interface of 37% and 70% Percoll, were collected. These cells were incubated with a mouse CD16/CD32 antibody (BD Biosciences) for 5 min and surface stained with PE‐labeled CD45 and FITC‐labeled CD11b antibodies or PE‐labeled CD45, APC‐labeled CD11b, and PE‐Cy7‐labeled Ly‐6C, as per manufacturer's instructions (BD Biosciences). CD45^int^/CD11b^+^ cells were then assessed and sorted on a Moflo XDP (Beckman Coulter). CX3CR1‐YFP^+^/CD11b^+^/CD45^int^/Ly‐6C^−^ cells were also assessed on BD LSRFortessa (BD Biosciences).

### Human MS Tissue Specimens

MS and control cases samples (wet weight/sample between 1 and 1.5 g) were kindly gifted by Netherlands Brain Bank. According to the record, unfixed whole brains (pH range, 6.22–7.08; mean, 6.50) from seven MS patients (age range, 40–77 years; mean age, 56 years) were obtained at autopsy under the management of the Netherlands Brain Bank within 10 h of death (mean postmortem delay, 6.8 h). Twenty‐five tissue blocks from seven different multiple sclerosis patients and seven tissue blocks from seven subjects without any known neurological disease were used for miRNA analyses. These formalin‐fixed, paraffin‐embedded multiple sclerosis tissue samples were selected on the basis of postmortem MRI. The control white matter specimens were from seven donors of non‐demented subjects. Multiple sclerosis lesions were characterized as described previously,^[^ ^81^ ^]^ using Luxol Fast Blue‐periodic acid Schiff (LFB‐PAS) staining. NAGM, normally appearing grey matter, consists of neuronal cell bodies and relatively few myelinated axons. NAWM is normally appearing white matter with intact myelin. Lesions with florid parenchymal and perivascular inflammatory cell infiltration, myelin fragmentation, and demyelination with indistinct margins were classified as acute active plaques. Chronic active plaques were classified as those with extensive demyelination, well‐demarcated borders, and abundant inflammatory cells at the lesion edge. Chronic inactive plaques were classified as those with extensive demyelination and well‐demarcated borders, but few or no inflammatory cells in any part of the demyelinated area.^[^ ^19^ ^]^ The collection and use of human tissue was approved by the Institutional Review Board of the SMMU, Shanghai, China (Li900).

### Cell Cultures

CNS mixed glial cell cultures were generated and cultured in DMEM/F‐12 containing 10% fetal bovine serum (D10; Invitrogen) and an antibiotic mixture (1% penicillin/streptomycin; Invitrogen) at 37 °C and 5% CO_2_ for 10 d. For the purification of microglia, cultures were shaken for 6 h at 180 rpm at 37 °C to collect microglia. HUVECs (ATCC PCS‐100‐010) were maintained on Corning tissue culture dishes (Corning Inc., Corning, NY) in a humidified atmosphere of 5% CO_2_, 95% air in DMEM (Hyclone.sh30243.01) with 10% fetal bovine serum as previously reported.^[^ ^82^ ^]^ BMVEC were isolated as reported.^[^ ^83^ ^]^ Briefly, after meninges had been detached, brain cortex was minced and digested in a mixture of 0.6 mL collagenase CLS2 (10 mg mL^−1^ in DMEM, Worthington, LS004176) and 0.2 mL DNAse (1 mg mL^−1^ in PBS, Worthington, LS002138) in DMEM. After the myelin had been removed, the pellet was then resuspended in 9 mL DMEM and 1 mL collagenase/dispase (final concentration, 1 mg mL^−1^) with 0.1 mL DNAse. After suspended, centrifuged, and resuspended, the digested cell suspension was added on top of 33% Percoll (17‐0891‐01, Amersham Pharmacia Biotech, Piscataway, NJ) gradient and centrifuged. The interphase with cells was acquired, centrifuged, and resuspended in BD Endothelial Culture Medium (containing the following components to the base medium: 0.1 mg mL^−1^ heparin; 0.03–0.05 mg mL^−1^ endothelial cell growth supplement [ECGS]). The cells were seeded, cultured, and split at 90% confluence. Purified BMVEC were identified by immunofluorescence staining for CD31 (Sigma‐Aldrich, P‐8590).

### Paracellular Permeability Assay

In vitro permeability was assayed using FITC‐dextran fluorescein.^[^ ^84^, ^85^ ^]^ The HUVECs were seeded onto 0.4‐µm pore 12‐well collagen‐coated Transwell inserts (Corning Costar Corp., Cambridge, MA) at 5 × 10^5^ per well and were cultured in BD Endothelial Culture Medium (BD Biocoat Endothelial Cell Growth Environment; BD Bioscience, San Jose, CA). The medium was replaced every second day with 1.5 mL to the outer and 0.5 mL to the inner chambers. When the cells reached confluence at day 5, they were incubated with microglia condition medium as indicated. At each time point, 50 µL of 12.5 mg mL^−1^ FITC‐dextran 4 (FD4, Sigma‐Aldrich, St. Louis, MO) was added to the inner chamber with final total volume of 0.5 mL. To determine the permeability of the cellular barrier, aliquots of 20 µL from the outer chamber were taken at each time point as indicated. Extravasated FITC‐dextran 4 was measured with excitation and emission wavelengths at 490 and 520 nm using NanoQuant Infinite M200 Pro (Tecan, Zurich, Switzerland). A standard curve was prepared with known concentrations of serially diluted FITC‐dextran 4. Fluorescein values were corrected for repetitive sampling.

### Transendothelial Electrical Resistance Analysis

TEER analysis was used to detect monocellular BBB permeability of cultured HUVECs and BMVECs, as reported.^[^ ^35^, ^86^ ^]^ Briefly, HUVECs or BMVECs were seeded at 200 000 cells cm^−2^ on collagen‐coated 24‐well transwell inserts (Corning) 72 h prior to the experiment. Cells were then serum‐starved for 4 h and stimulated with the microglial condition medium as indicated. TEER was measured at the indicated time points using a chopstick electrode with an EVOM2 meter (World Precision Instruments, FL, USA). Then, blank TEER values, measured without cells in transwell inserts, were subtracted. Resistance per cm^2^ was calculated and normalized to untreated control cells at the start of stimulation (0 h). Results were depicted as the steady‐state TEER values with blank filter subtracted.

### Protein Array Assay

Culture medium of microglia was collected following an incubation time of 24 h and filtered through a 20 µm mesh (BD Falcon). Antibody arrays (Mouse L308 Array; RayBiotech AAM‐BLG‐1‐2, GA, 30092) were used to profile the proteins from microglia supernatant in response to miR‐126a‐5p overexpression. Each protein was examined in triplicates and the means for each of the 308 proteins were compared between the miR‐126a‐5p mimics and the NC mimics groups. Array analysis was conducted as per manufacturer's instructions (Raybiotech, Guangzhou, China). Briefly, Proteins were clicked with Biotin‐Label Based G Series. Nanodrop was used to quantify the clicked product. The arrays were detected with Cy3 labeled streptavidin, as recommended by the manufacturer. Arrays were scanned using InnoScan 300 Microarray Scanner (Parc d'activités Activestre, Carbonne, France) at 532 nm. For each protein, duplicate spots on each array were averaged, local background fluorescence was subtracted and resulting fluorescence signal was normalized by the internal positive controls on the arrays. The basic statistics used for significance analysis are moderated *t*‐statistic. Results include fold changes and *p*‐values for each protein and for each contrast individually. Differentially expressed proteins (DEPs) are defined as those with *p*‐value less than 0.05, and foldchange over 1.2 or less than 0.83.

### Viral Infection and MiRNA Mimics Transfection

The coding sequence of miR‐126a‐5p (miR‐126a‐5p‐OE) (MIMAT0000137) or GFP alone (Ctrl‐OE) was ligated into the GV287 plasmid (GeneChem). The siRNAs for miR‐126a‐5p (miR‐126a‐5p‐i) or GFP alone (Ctrl‐i) were ligated into the GV280 plasmid (GeneChem). The sequences for miR‐126a‐5p siRNA were as follows: 5′‐AAUUCUUUUUCAUUAUUACUUUUGGUACGCG‐3′. Titers of concentrated viral particles ranged between 1 × 10^8^ and 2 × 10^9^ transducing units mL^−1^. Lentiviral particles were added on day 2 to cultured microglia. The supernatant was removed 24 h after infection and replaced with D10 medium. The target sequences in mmp9 (GeneBank, NM_013599) chosen from six candidates for RNAi was as follows: 5'‐ CCCACTATGTGTCCCACTATA‐3'. A pair of primers containing the target sequence and sticky ends were annealed and ligated into the linearized vector WX647(AAV2/9‐hEF1a‐DIO‐mCherry) (Taitool Bioscience, Shanghai, China.), using the following primers: 5′‐TGCTGTATAGTGGGACACATAGTGGGGTTTTGGCCACTGACTGACCCCACTATGTCCCACTATA‐3′, 5'‐ CCTGTATAGTGGGACATAGTGGGGTCAGTCAGTGGCCAAAACCCCACTATGTGTCCCACTATAC ‐3'. Then the expression plasmids were co‐transferred with Ad Helper and RepCap plasmids into 293T cells. Purification of the AAV2/9‐hEF1a‐DIO‐Mmp9‐miRNAi‐mCherry was completed through CsCl density‐gradient centrifugation. Titers of concentrated viral particles was 1.65 to 1.95 × 10^13^ V.G. mL^−1^. For Cre‐driven AAV virus transduction to interfere with mmp9, AAV virus to interfere with mmp9 (AAV‐DIO‐MMP9‐i) (Taitool Bioscience, Shanghai, China.) or control (AAV‐DIO‐Ctrl‐i) (S0597‐9, Taitool Bioscience, Shanghai, China) were injected into CX3CR1‐126CKO mice intrathecally 14 d before MOG immunization (Figure 5D).

For transfection of microglia with miRNA mimics, 200 × 10^−9^ m of miR‐126a‐5p mimics (5′‐CAUUAUUACUUUUGGUACGCG‐3′) or control mimics (miRNA‐NC) (5′‐UUUGUACUACACAAAAGUACUG‐3′) was used as per manufacturer's recommendation (RiboBio, Guangzhou, China).

### MiRNA‐Seq

Total RNA was extracted from FACS sorted microglia in spinal cord from EAE mice at indicated time points using a miRNeasy Mini Kit (QIAGEN 217004, Germany). RNA quality was evaluated with a BioAnalyzer 2100 system (Agilent Technologies). Small RNAs had linkers ligated to them and bar‐coded cDNAs were prepared using a NEB Next Multiplex Small RNA Library Prep Set for Illumina (New England Biolabs, Ipswich, MA, E7580S) following the manufacturer's instructions. Individual libraries were analyzed for the presence of linked cDNA at the appropriate size (140–150 bp) as determined by the BioAnalyzer 2100 system (Agilent, Santa Clara, CA). Subsequently, the amplified cDNA constructs were purified from agarose gel in preparation for sequencing analysis using the Illumina Xten platform (Illumina, San Diego, CA) as per manufacturer's instructions at the Shanghai Biotechnology. The small RNA sequence reads were preprocessed using the FASTX Toolkit (version: 13) to exclude low‐quality reads (ambiguous N, 3'*Q* < 10 (*Q* = −10log error ratio), and length < 18 nt) and 3' adapter, 5' adapter and poly(A) sequences. The remaining high quality reads were aligned to the miRBase (version 21.0) reference sequence and the mouse genome (GRCm38). After all annotation steps, the sequencing libraries were used for size distribution and saturation analysis. For the co‐expression analysis between BBB disruption (density of EVB) and expression pattern of miRNA (|R| > 0.95 and *p* < 0.05) and the TOP 50 in the abundance hierarchies of miRNA, please see in Table S1 (Supporting Information).

### FISH

The LNA microRNA probe that was used for FISH was purchased from EXIQON, as previous reported.^[^ ^45^, ^87^ ^]^ The TSA‐Plus Fluorescein System was purchased from PerkinElmer.

### Luciferase Assay

As reported previously,^[^ ^88^ ^]^ a dual luciferase reporter system was used in this work and the binding between miR‐126a‐5p and the 3' UTR of mmp9 was shown by a decrease in the fluorescence intensity. The 3' UTR of mmp 9 or a mutated 3' UTR of mmp9 (mut UTR) was cloned in pGL3‐basic vector. The binding between miR‐126a‐5p and the 3' UTR of mmp9 linked to the mRNA of luciferase impeded the translation of luciferase. HEK‐293 T cells were co‐transfected with the luciferase reporter gene construct, the pRL‐SV40 Renilla luciferase construct, and miR‐126a‐5p mimics, mutate miR‐126a‐5p mimics (Mut miRNA) or negative control mimics (miRNA‐NC) (RiboBio, Guangzhou, China). Cell extracts were prepared 24 h after transfection and the luciferase activity was measured with the Dual‐Luciferase Reporter Assay system (Promega, 017319). The miRCUYR LNA microRNA Power Inhibitors (TP) (Exiqon, Denmark) was custom‐designed for competing the miR‐126a‐5p effects on mmp9. The sequences were indicated as follows:

TP: 5′‐CGTGTTTATTAGAAACAGT‐3′; TP‐NC: 5′‐GCTCCCTTCAATCCAATATAATCA‐3′.

### RNA Isolation and QPCR

As described previously,^[^ ^89^ ^]^ total RNA was extracted from spinal cord or from the primary cell culture using Trizol (Invitrogen). First‐strand cDNA was synthesized using a RevertAid First Strand cDNA Synthesis kit (Thermo Fisher Scientific, Fermentas) or Taqman MicroRNA Reverse Transcription Kit (Thermo Fisher Scientific, Vilnius, Lithuania). Quantitative PCR was performed on a LightCycler 480 Real Time PCR system (Roche). Gene expression was expressed as the miRNA level, which was normalized to that of a standard housekeeping gene (Rnu6 or snoRNA202) using the ΔΔCT method. Primer pairs are listed in Table S5 (Supporting Information).

### Detection for Cytokines, ROS, and NO

All samples were frozen at −80 °C until all biological replicates have been acquired. Then, detection for Cytokines, ROS and NO were performed immediately. The concentration of TNF‐*α* (R&D Systems, MN), MMP9 (Boster, Wuhan, China), and IL‐1*β* (R&D Systems, MN) were detected using an ELISA as per manufacturer's instructions. Absorbance was read at 490 nm on a spectrophotometer, and sample concentrations were calculated using an equation generated from a standard curve. ROS was determined by FACS using the total ROS Assay Kit 520 nm (Affymetrix, 88‐5930) as per manufacturer's instruction. NO was measured by nitrate reductase method using NO detection kit (Jiancheng Bioengineering, Nanjing, China). Briefly, the absorbance was read at 550 nm on a spectrophotometer, and the content of NO_2_^−^ is determined colorimetrically.^[^ ^90^ ^]^

### Immunofluorescence Staining

Cells or tissue sections were fixed, permeabilized, and incubated with primary antibodies [Iba1 (wako), laminin (Boster), CD31 (Boster, Abcam, HuaBio), albumin (Invitrigen), YFP (SAB), ZO‐1 (Proteintech), mCherry (Abcam), MMP9 (Boster), Aqp4 (Abcam), PDGFR*β* (Abcam)] overnight at 4 °C, followed by incubation with TRITC‐conjugated, Alexa647‐conjugated, or FITC‐conjugated secondary antibody (Jackson ImmunoResearch) and counterstained with Hoechst33342 (Sigma‐Aldrich). Fluorescence images were captured using fluorescence microscopy (DXM1200, Nikon) or confocal microscope (TCS SP5, Leica) and quantified using Image‐Pro Plus (Media Cybernetics) or Image J (NIH Image, Bethesda, MD).^[^ ^45^ ^]^

### Immunoblot Analysis

Primary cell cultures were homogenized in RIPA buffer supplemented with protease cocktail inhibitors (Roche). Cell lysates were subjected to Western blot analysis using ZO‐1 (Proteintech), OCLN (Invitrigen, Life tech), and MMP9 (Boster) antibody. The protein bands were acquired and analyzed using a ChemiDoc XRS System with ImageLab software (Bio‐Rad, Hercules, CA), normalizing indicated protein to GAPDH bands by Image‐Pro Plus (Media Cybernetics).

### Hematoxylin and Eosin and Luxol Fast Blue Staining

H&E or Luxol fast blue with periodic acid Schiff (LFB‐PAS) were performed as previously described. Lumbar or thoracic spinal cords were isolated and cut into cryosections (7–14 µm thick) for immunohistochemistry. One of every six successive sections was collected from each animal. One pool of the sections was stained with LFB‐PAS. Another pool of the sections was stained with H&E solution and mounted in Permount (Fisher Scientific). Scoring for LFB‐PAS or H&E sections was performed using blind examination by independent readers.

### Transmission Electron Microscopy

As previously described,^[^ ^45^ ^]^ spinal cords of mice were fixed in 2.5% glutaraldehyde for 2 h, postfixed in 1% osmium tetroxide for 45 min, dehydrated, and embedded in Araldite resin. Sections (1 µm) were stained with toluidine blue. Ultrathin sections (60 nm) were stained in uranyl acetate and lead citrate. Samples were visualized using a transmission electron microscope (H‐7650; Hitachi) at 100 kV.

### Drug Screen

640 compounds of FDA approved drug from BML‐2842 (v1.2) Library (10 × 10^−3^ m in DMSO, National Compound Resource Center, Shanghai, China) were diluted in DF12. For drug screen, miR‐126a‐5p mRNA was measured by qPCR in microglia treated with 10 × 10^−6^ m of each indicated compound (Table S3, Supporting Information) for 24 h. DMSO treatment was used as a control. For validation, mmp9 mRNA level was determined by qPCR in microglia treated with each selected compound (Table S4, Supporting Information). The investigators were blinded to allocation of compounds during the screening until the final statistical analysis.

### Statistics Analyses

They were performed using GraphPad Prism software (version 7.02). The univariate of normality assumption was verified with Shapiro–Wilk tests on the error distribution from the statistical model. The Brown and Forsythe variation of the Levene test statistic was used to verify the homogeneity of variances. When distributions were not normal, or requirements for parametric tests were not met, nonparametric comparisons (Mann–Whitey test or Kruskal–Wallis test with post hoc test) were performed. Otherwise, the following statistical tests were used as indicated: Student's *t*‐test, one‐way ANOVA, and two‐way ANOVA, followed by post hoc test. The data are presented as the mean ± SEM unless otherwise indicated. *P* < 0.05 was considered statistically significant.

## Conflict of Interest

The authors declare no conflict of interest.

## Author Contributions

Z.Y., X.F., and W.L. contributed equally to this work. Z.Y., L.C., and C.H. conceived the study. Z.Y., X.F., and W.L. performed most of the experiments and analyzed the data. M.Z. and Y.P. assisted with drug screen and animal experiments. Z.X. contributed to FISH. D.S., P.L., and Y.D. helped design some of the experiments and contributed to discussion. Z.Y., L.C., and C.H. wrote the manuscript.

## Supporting information

Supporting Information

Supporting Table 1

Supporting Table 2

Supporting Table 3

Supporting Table 4

Supporting Table 5

## Data Availability

The data that support the findings of this study are available from the corresponding author upon reasonable request.
