# Supplementary material for: Microglia Regulate Blood–Brain Barrier Integrity via MiR‐126a‐5p/MMP9 Axis during Inflammatory Demyelination
Source: Adv Sci (Weinh). 2022 Jun 27;9(24):2105442. doi: 10.1002/advs.202105442 (PMC9403646; doi:10.1002/advs.202105442)
Supplement: Supplementary file 1 — Supporting Information [file ADVS-9-2105442-s001.pdf]

ADVANCED SCIENCE
Open Access

Supporting Information

for *Adv. Sci.*, DOI 10.1002/advs.202105442

Microglia Regulate Blood–Brain Barrier Integrity via MiR-126a-5p/MMP9 Axis during Inflammatory Demyelination

Zhongwang Yu, Xue Fang, Weili Liu, Rui Sun, Jintao Zhou, Yingyan Pu, Ming Zhao, Dingya Sun, Zhenghua Xiang, Peng Liu, Yuqiang Ding, Li Cao and Cheng He**

Supporting Information

Microglia Regulate Blood-Brain Barrier Integrity via MiR-126a-5p/MMP9 Axis during Inflammatory Demyelination

Zhongwang Yu, Xue Fang, Weili Liu, Rui Sun, Jingtao Zhou, Yingyan Pu, Ming Zhao, Dingya Sun, Zhenghua Xiang, Peng Liu, Yuqiang Ding, Li Cao, and Cheng He **

This file includes:

- Figure S1 to 10
- Captions for Table S1 to 5

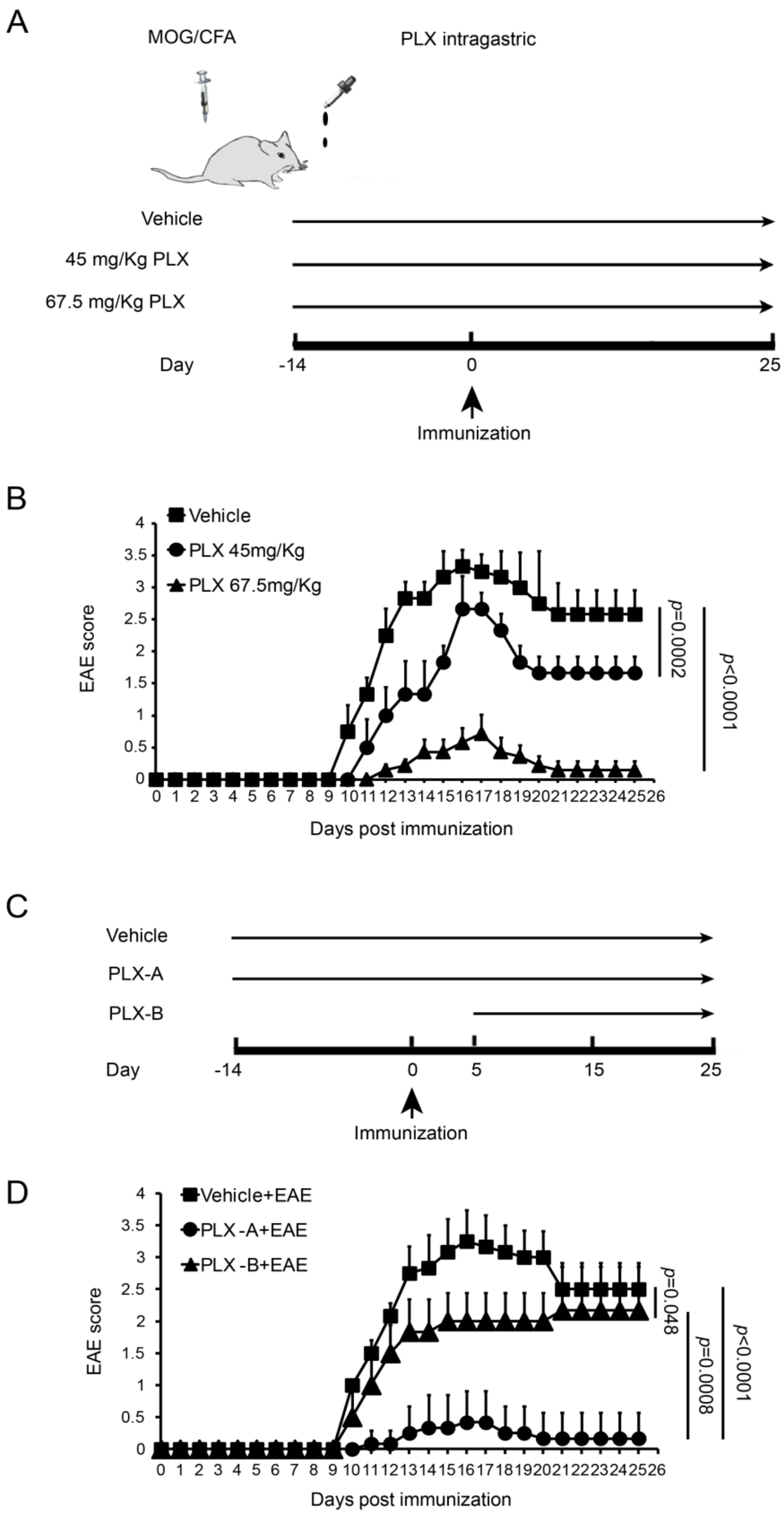

Figure S1. Ablation of microglia ameliorates EAE progression. (A) Scheme of microglial ablation and time points for immunization and examination. **(B)** The clinical score of EAE administrated with different dosages of PLX5622 or vehicle

daily from D-14 to D25 post MOG-immunization. **(C)** Scheme of microglial ablation and time points for immunization and examination. **(D)** The clinical score of EAE administrated with PLX5622 or vehicle as indicated. Kruskal-Wallis test with Dunnett's multiple comparisons test was used in (B, D). N = 6 mice per group for EAE score analysis. Data are shown as the mean ± SEM.

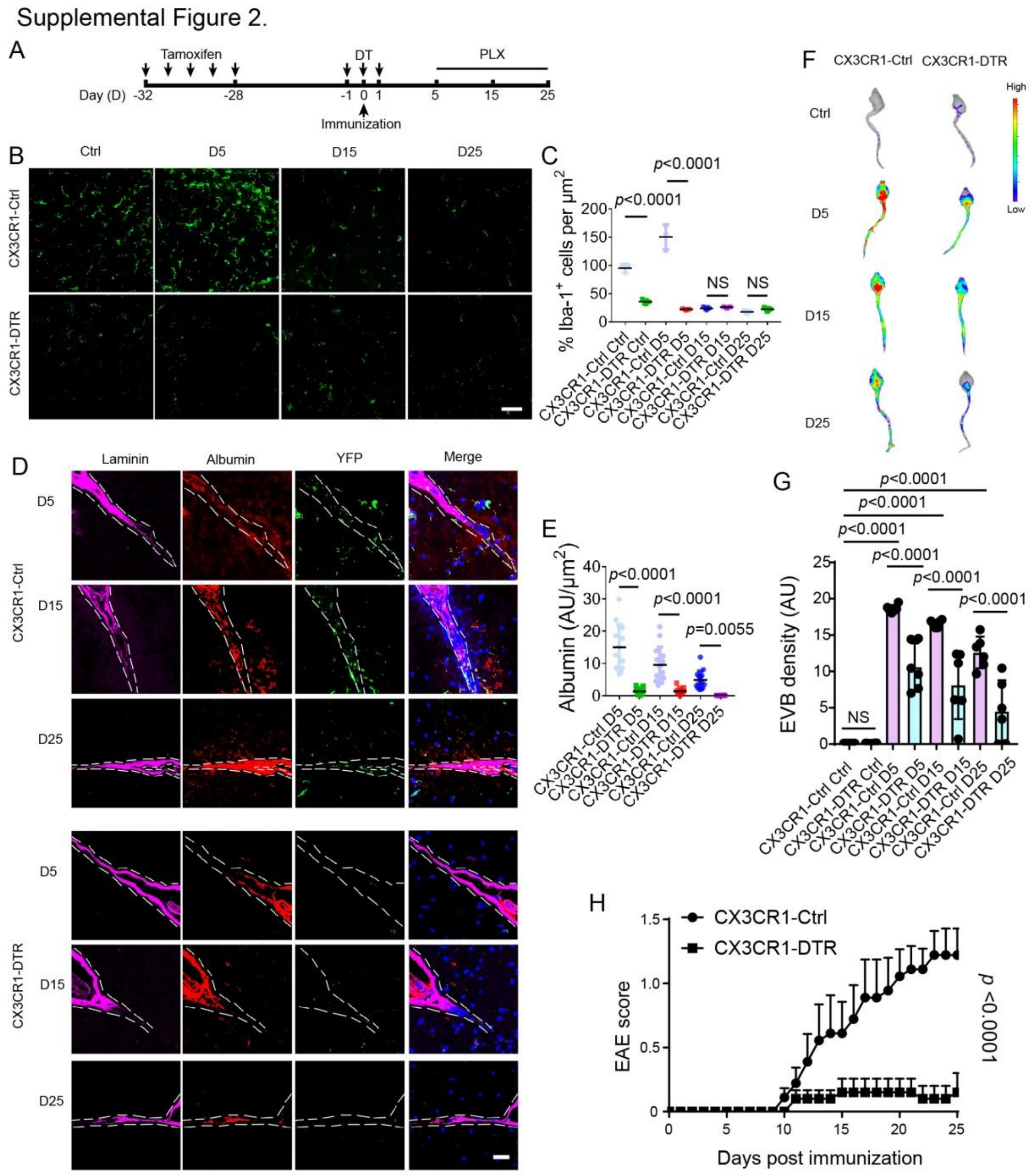

Figure S2. Ablation of microglia in CX3CR1-DTR mice attenuates BBB disruption and ameliorates EAE progression. (**A**) Scheme of microglial ablation and time points for immunization. (**B, C)** Representative images show Iba-1^{+} cells (green) in spinal cord sections of CX3CR1^{CreER} (CX3CR1-Ctrl) or CX3CR1^{CreER}: iDTR (CX3CR1-DTR) mice with EAE at different stages. N = 3 mice per group. (**D, E**) Temporal dissemination of the BBB in the lumbar spinal cord from EAE mice.

Representative images of YFP (green), Albumin (red), Laminin (magenta, Alexa Fluor 647) and cell nuclei (blue) at different stages of EAE. The dashed lines indicate the boundary of Laminin^{+} vessels. Note the YFP^{+} microglia congregate around the albumin beyond the border of leaky vessels from D5 to D25 in CX3CR1-Ctrl group, whereas no YFP^{+} microglia are observed in CX3CR1-DTR group. N = 3 mice per group. (**F, G**) Representative images show BBB integrity in CX3CR1-Ctrl or CX3CR1-DTR mice with EAE after *i.v.* EVB injection by optical imaging. N = 3 mice per group. (**H**) The clinical score of EAE. N = 6 mice per group. Scale bars: 50 μm. One-way ANOVA with a Holm–Sidak's multiple comparisons test is used in (C, E, G). Mann-Whitney test is used in (H). Data are shown as the mean ± SEM.

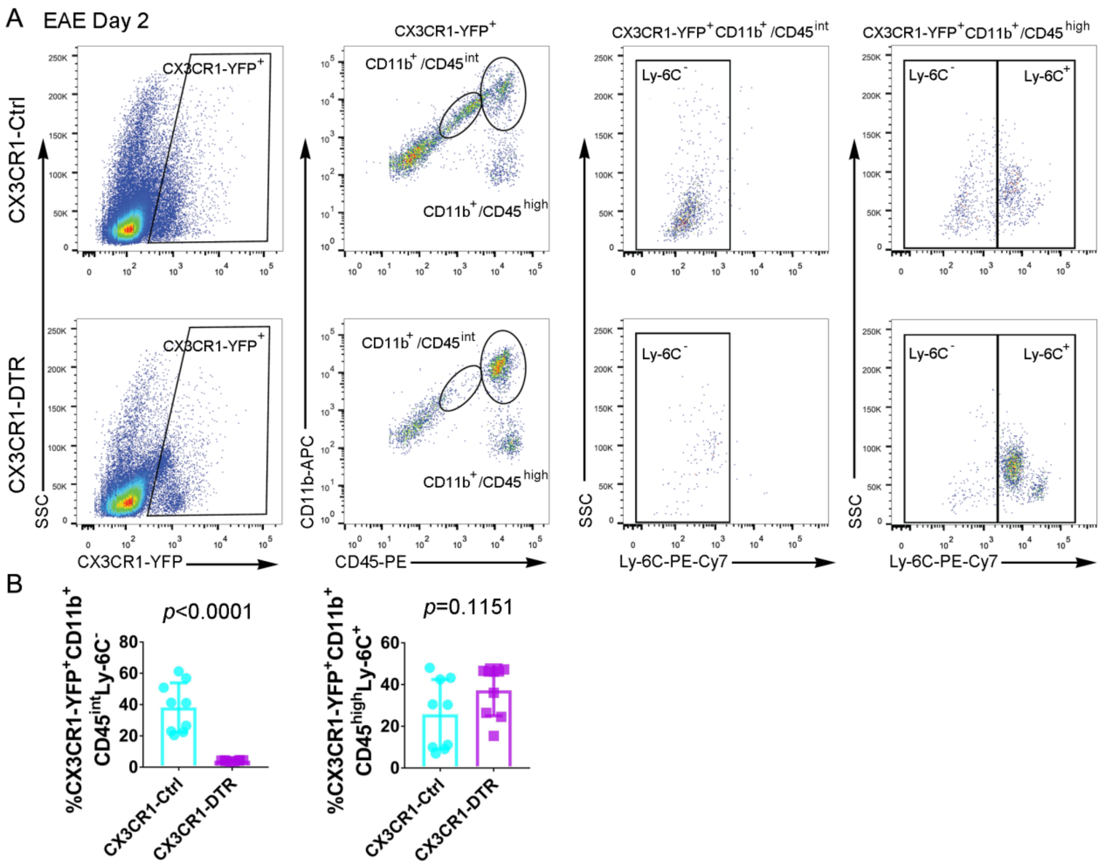

Figure S3: Microglia but not peripheral immune cell ablation is confirmed 24hrs after DT injection (EAE D2). (A) Microglia and peripheral immune cell infiltrates are distinguished and sorted as CX3CR1-YFP^{+} CD11b^{+} CD45^{int} Ly-6C^{-} and CX3CR1-YFP^{+} CD11b^{+} CD45^{high} Ly-6C^{+}, respectively. FACS plots of a representative mouse are shown of a total of 3 mice per condition. (B) The percentage of sorted microglia and peripheral immune cells are illustrated. Unpaired Student's t-test is used. N = 9 per group. Data are shown as the mean ± SEM.

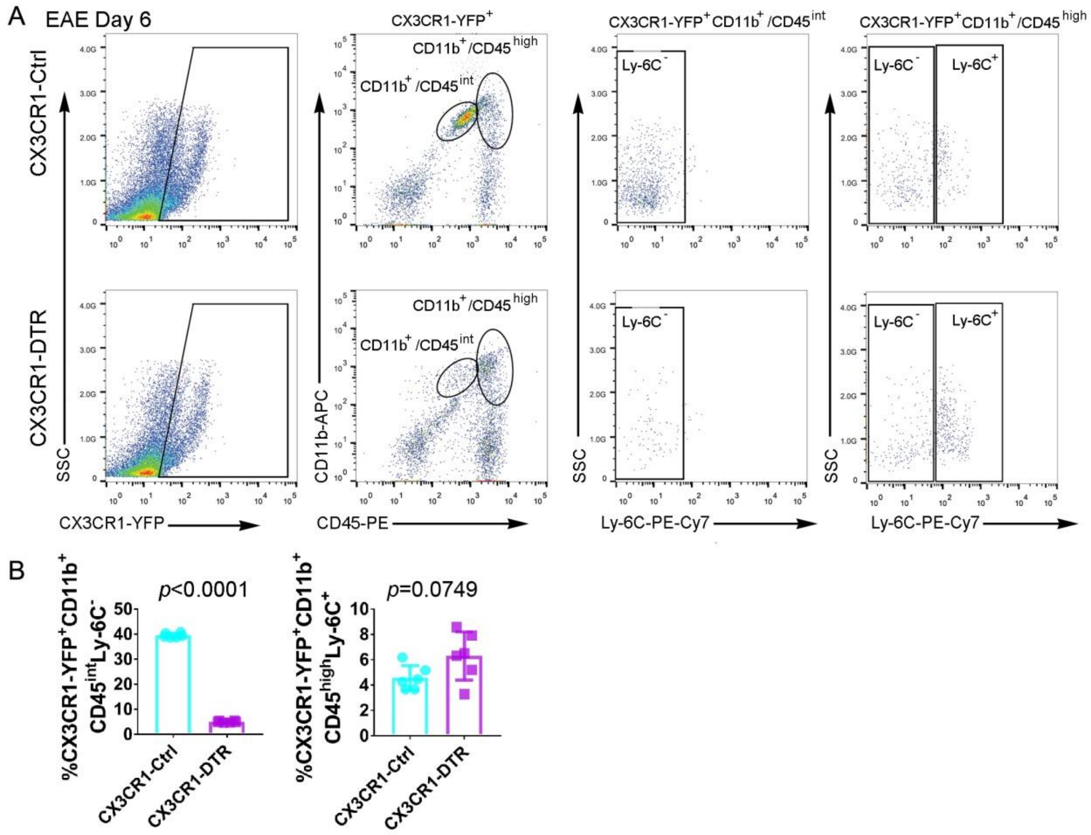

Figure S4: Microglia but not peripheral immune cell ablation is confirmed 24hrs after PLX-treatment (EAE D6). (A) Microglia and peripheral immune cell infiltrates are distinguished and sorted as CX3CR1-YFP^{+} CD11b^{+} CD45^{int} Ly-6C^{-} and CX3CR1-YFP^{+} CD11b^{+} CD45^{high} Ly-6C^{+}, respectively. FACS plots of a representative mouse are shown of a total of 3 mice per condition. (B) The percentage of sorted microglia and peripheral immune cells are illustrated. Unpaired Student's t-test is used. N = 6 per group. Data are shown as the mean ± SEM.

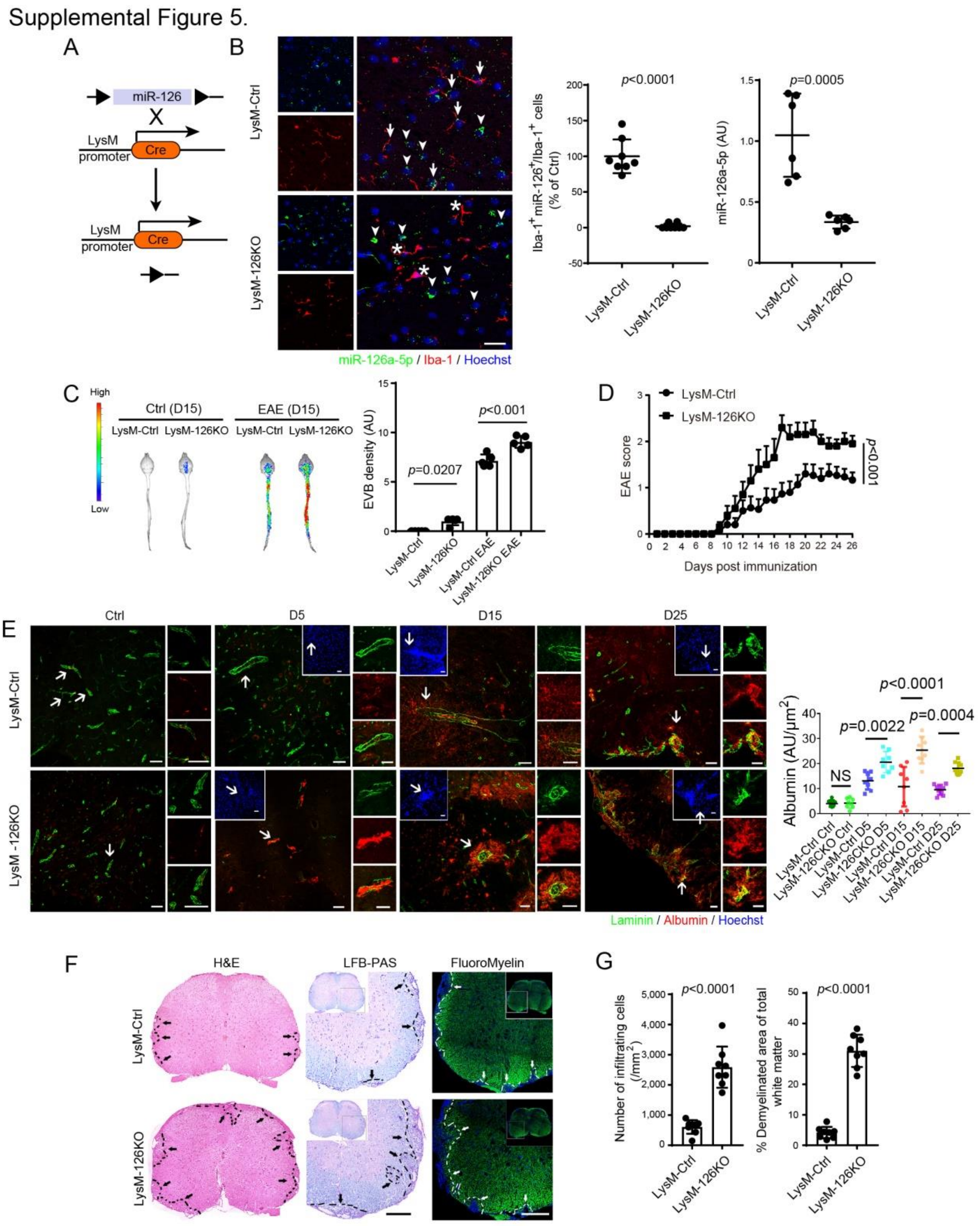

Figure S5. Ablation of miR-126a-5p in myeloid cell increases BBB leakage and exacerbates EAE progression. (**A**) Schematic graph of generation of mice to delete miR-126a-5p specifically in myeloid cell. (**B**) The miR-126 expression in myeloid cell is detected by FISH and QPCR. Arrows indicate Iba1^{+} miR-126a-5p^{+} cells. Arrow

heads indicate Iba1$^-$ miR-126a-5p$^+$ cells. Stars indicate Iba-1$^+$ miR-126a-5p$^-$ cells. Scale bars: 50 μm. FISH: N = 8 per group. QPCR: N = 6 per group. (**C**) Representative images show BBB integrity in control (LysM^{Cre}:126$^{+/+}$, LysM-Ctrl) or miR-126a-5p knockout (LysM^{Cre}:126$^{fl/fl}$, LysM-126KO) mice with or without EAE on D15 after *i.v.* EVB dye injection by optical imaging. One-way ANOVA with a Tukey's multiple comparisons test is used. N = 5 in each group. (**D**) Clinical score of EAE mice. Mann–Whitey test is used. N = 10 for LysM-126KO mice. N = 15 for LysM-Ctrl mice. (**E**) Representative images and quantitative analysis of Albumin. Albumin (red) leaked through vessel (Laminin, green) in the sections of spinal cord of EAE mice. Arrow indicates boxed area in the right column. Scale bars: 50 μm. One-way ANOVA with a Holm–Sidak's multiple comparisons test is used. N = 2 mice at each time point per group. (**F**) Representative spinal cord sections from EAE mice after H&E, LFB-PAS and fluoromyeline staining. Scale bars: 200 μm. (**G**) Quantification of cell infiltration and demyelination. Unpaired Student’s t-test is used in (B) and (G). N = 8 per group in (G). Data are shown as the mean ± SEM.

E 中 N=2 mice 是否正确？

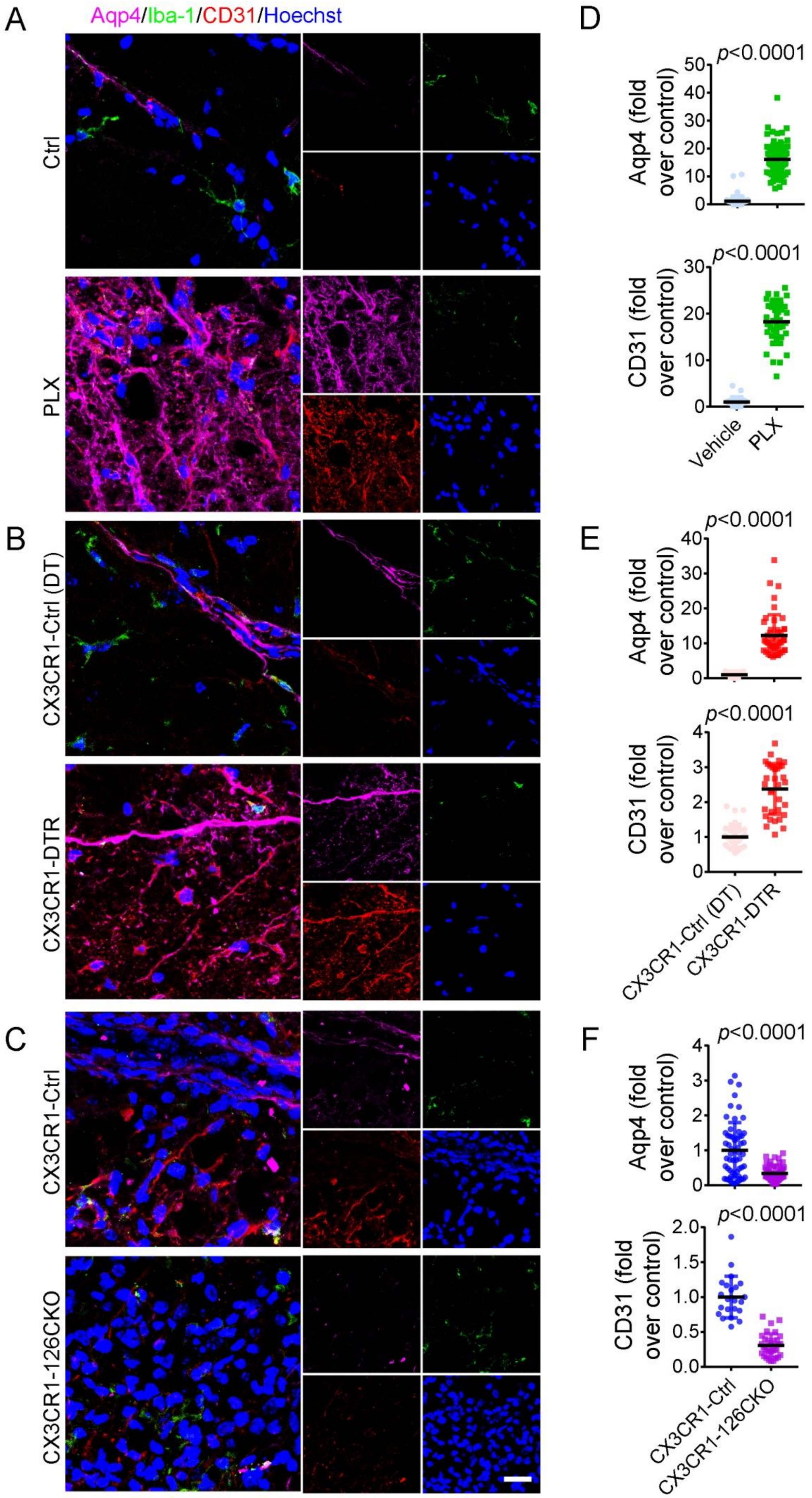

A
Aqp4/Iba-1/CD31/Hoechst
Ctrl
PLX
B
CX3CR1-Ctrl (DT)
CX3CR1-DTR
C
CX3CR1-Ctrl
CX3CR1-126CKO
D
p<0.0001
Aqp4 (fold over control)
CD31 (fold over control)
Vehicle
PLX
E
CX3CR1-Ctrl (DT)
CX3CR1-DTR
F
CX3CR1-Ctrl
CX3CR1-126CKO

Figure S6. Microglial ablation attenuates astrocyte endfeet disruption, promotes endothelial activation, whereas ablation of miR-126a-5p in microglia aggravates deficits of astrocytes and endothelial cells in EAE. (**A-C**) Representative images of Aqp4 (magenta, Alexa Fluor 647), Iba-1 (green), CD31 (red) and cell nuclei (blue) in lumbar spinal cord sections of PLX5622 treated (A), CX3CR1-DTR (B) or microglia miR-126a-5p conditional knockout mice (C) with EAE at D15. Scale bars: 50μm. (D-F) Quantitative analysis of relative intensity of astrocytic endfeet densities (Aqp4) and vascular densities (CD31). N= 3 mice per group. Mann-Whitney test is used in (D-F). Data are shown as the mean ± SEM.

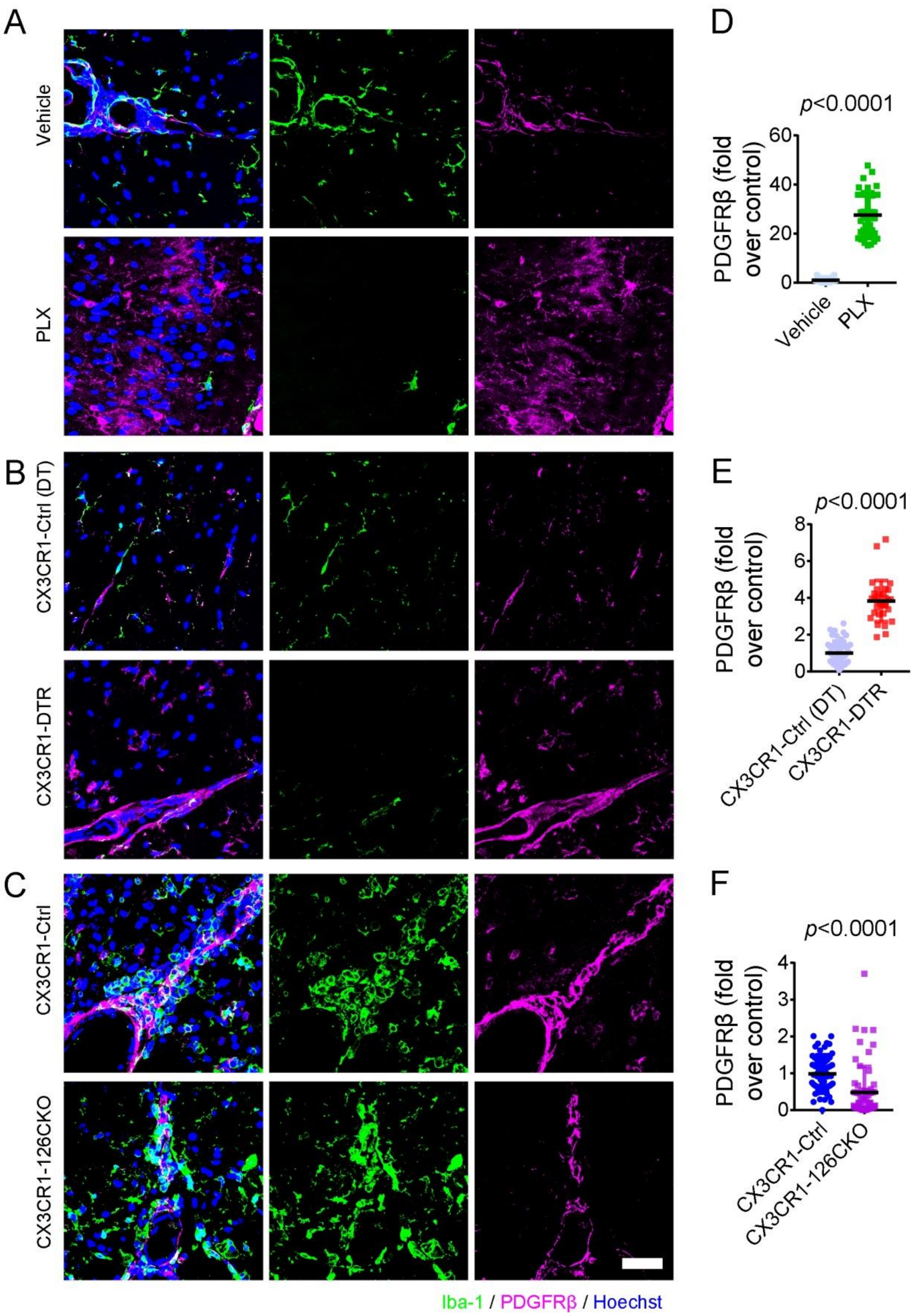

Figure S7. Microglial ablation attenuates deficits of pericytes, whereas ablation of miR-126a-5p in microglia aggravates it in EAE. (**A-C**) Representative images of PDGFRβ (magenta, Alexa Fluor 647), Iba-1 (green) and cell nuclei (blue) in lumbar

spinal cord sections of PLX5622 treated (A), CX3CR1-DTR (B) or microglia miR-126a-5p conditional knockout mice (C) with EAE at D15. Scale bars: 50 μm. (D-F) Histology analysis of the extent of pericyte densities. N=3 mice per group. Mann-Whitney test is used in (D-F). Data are shown as the mean ± SEM.

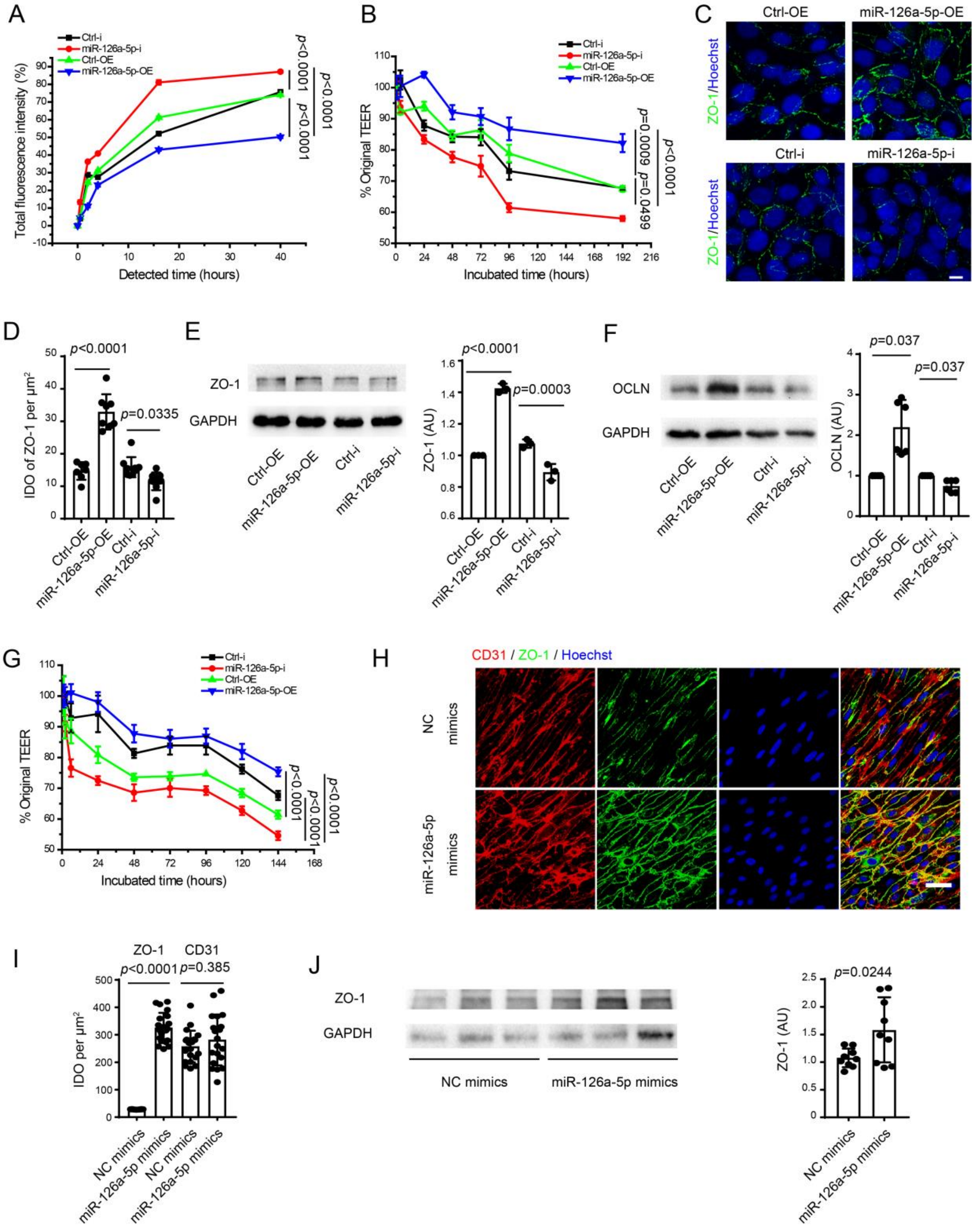

Figure S8. The miR-126a-5p in microglia is crucial for preserving the barrier

integrity of HUVECs and BMVECs. (**A**) The permeation rate of FITC-dextran fluorescein (FD40) through HUVECs in transwell inserts with CM from microglia infected with lentivirus (miR-126a-5p-i or miR-126a-5p-OE). N = 3 per group. (**B**) TEER of the endothelial monolayer cultured with different microglial CM. N = 4 per group. (**C**) Representative images show the barrier integrity of HUVECs treated with different microglial CM. Scale bar: 10 μm. (**D**) Quantitative analysis of relative intensity of ZO-1. N ≥ 7 area per group. IOD, Integrated optical density. (**E**, **F**) Immunoblot and densitometric analyses of ZO-1 and OCLN in HUVECs incubated with different microglial CM. N ⩾ 3 per group. (**G**) TEER of BMVECs with different microglial CM. N = 3 per group. (**H**, **I**) Representative images (H) and quantitative analysis (I) of relative intensity of ZO-1 and CD31 of BMVECs incubated with CM from microglia treated with miR-126a-5p mimics or control (NC mimics). N = 20 area per group. Scale bar: 50 μm. (**J**) Immunoblot and densitometric analyses of ZO-1 in BMVECs incubated with indicated microglial CM. N = 9 per group. One-way ANOVA with a Tukey's multiple comparisons test is used in (D, E); Kruskal-Wallis test with Dunn's multiple comparisons test is used in (F); Two-way RM ANOVA with a Tukey's multiple comparisons test is used in (A, B, G); One-way ANOVA with a Holm–Sidak's multiple comparisons test is used in (H, I); Unpaired Student’s t-test is used in (J). Data are shown as the mean ± SEM.

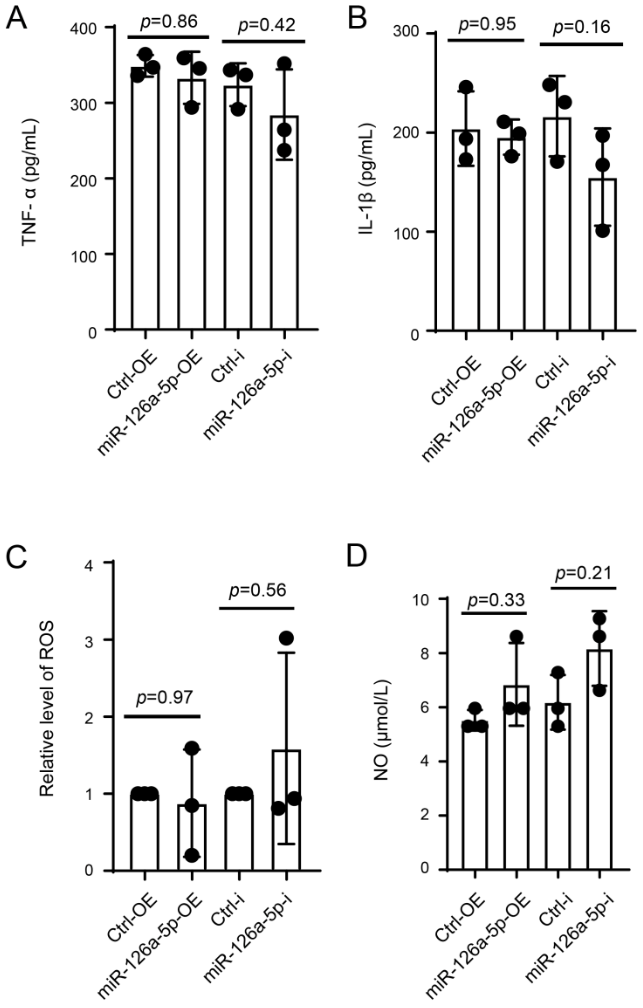

Figure S9. The levels of TNF-α (A), IL-1β (B), ROS (C) and NO (D) in indicated microglia detected by ELISA, ROS assay or NO assay, respectively. One-way ANOVA with a Holm–Sidak's multiple comparisons test is used in (A, B). Kruskal-Wallis test with Dunn's multiple comparisons test is used in (C, D). N = 3 per group. Data are shown as the mean ± SD.

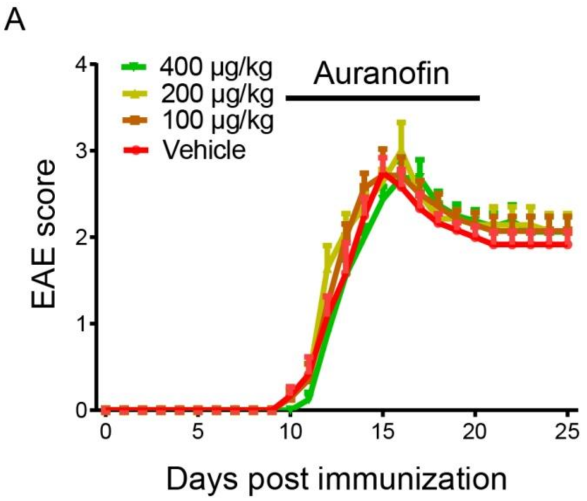

Figure S10. Treatment with Auranofin daily from D10 to D20 failed to ameliorate EAE severity. No significant differences of EAE score were observed among groups. Kruskal–Wallis test with Dunn's multiple comparisons is used. N = 7 mice per group. Data are shown as the mean ± SEM.

Table S1. miRNAs profile analysis of FACS sorted microglia.

Table S2. Protein array assay using LysM-126KO microglia transfected with miR-126a-5p mimics or NC mimics.

Table S3. List of compounds upregulating miR-126a-5p expression in microglia.

Table S4. List of compounds downregulating mmp9 expression in microglia.

Table S5. Primer pairs used in Quantitative PCR.
